# A scoping review, mapping, and prioritisation process for emergency obstetric and neonatal quality of care indicators: Focus on provision and experience of care

**DOI:** 10.7189/jogh.13.04092

**Published:** 2023-10-13

**Authors:** Dee Wang, Emma Sacks, Osamuedeme J Odiase, Ntemena Kapula, Alisha Sarakki, Erica Munson, Patience A Afulani, Jennifer Requejo, Revisioning Emergency Obstetric and Newborn Care (EmONC) quality of care workstream, Andreea Creanga, Alison Morgan, Allisyn Moran, Blerta Maliqi, Caitlin Warthin, Catherine Breen Kamkong, Dilys Walker, Jean-Pierre Monet, Kathleen Hill, Lenka Benova, Moise Muzigaba, Louise Tina Day, Patricia Bailey, Samantha Lobis, Sodzi Sodzi-Tettey, Tedbabe Degefie Hailegebriel

**Affiliations:** 1 Division of Data Analytics, Planning and Monitoring, United Nations Children’s Fund, New York, New York, USA; 2 Department of Population and Family Health, Columbia University Mailman School of Public Health, New York, New York, USA; 3 Department of International Health, Johns Hopkins Bloomberg School of Public Health, Baltimore, USA; 4 Institute for Global Health Sciences, University of California San Francisco, San Francisco, California, USA; 5 Department of Epidemiology and Biostatistics, University of California San Francisco, San Francisco, California, USA

## Abstract

**Background:**

Globally, approximately 800 women and 6400 newborns die around the time of childbirth each day. Many of these deaths could be prevented with high-quality emergency obstetric and newborn care (EmONC). The Monitoring Emergency Obstetric Care: A handbook guides strengthening EmONC services. However, the handbook contains limited quality of care measures. Our study identified and prioritised quality of care indicators for potential inclusion in the handbook, which is undergoing revision.

**Methods:**

We conducted a consultative scoping review, mapping, and prioritisation exercise to select a short list of indicators on facility-based maternal and newborn quality of care. Indicators were identified from literature searches and expert suggestions and organised by the categories of structure, process, and outcomes as defined in the World Health Organization’s Standards for Improving Quality of Maternal and Newborn Care in Health Facilities. We focused on process indicators, encompassing the provision of care and experience of care during the intrapartum period, and developed a priority list of indicators using the selection criteria of relevance and feasibility. Experience of care indicators were also mapped against the Person-Centered Maternity Care (PCMC) scale.

**Results:**

We extracted a total of 3023 quality of care indicators. After removing out-of-scope and duplicate indicators and applying our selection criteria, we identified 20 provision of care indicators for possible inclusion in the revised EmONC handbook. We recommend including a score for experience of care that could be measured with the 30-item or the 13-item PCMC scale. We also identified 29 experience of care items not covered by the PCMC scale that could be used. Provider experience, patient safety, and quality of abortion care were identified as areas for which no or few indicators were found through our scoping review.

**Conclusions:**

Through a rigorous, consultative, and multi-step process, we selected a short list of process-related, facility-based quality of care indicators for emergency obstetric and newborn care. This list could be included in the EmONC handbook or used for other monitoring purposes. Country consultations to assess the utility and feasibility of the proposed indicators and their adaptation to local contexts will support their refinement and uptake.

**Registration:**

https://osf.io/msxbd (Open Science Framework)

Although great progress has been made over the past two decades in decreasing maternal and newborn mortality, approximately 800 women and 6400 newborns still die each day around the time of childbirth [1,2]. Most of these deaths occur in low-resource settings and are preventable with high-quality, timely care. A key strategy to reduce maternal and newborn mortality is to improve the availability, accessibility, and use of emergency obstetric and newborn care (EmONC) services. However, this strategy will not result in expected maternal and neonatal mortality reductions if these services are of poor quality. Estimates suggest that each year, approximately one million newborn deaths and half of all maternal deaths could be averted if health systems were strengthened to deliver high-quality care [3]. Improvements in the quality of maternal health services could also reduce the estimated 1.9 million stillbirths that occur every year [2].

Numerous reports and initiatives in recent years have highlighted the centrality of high-quality services for ensuring positive obstetric outcomes [3-5]. Regular monitoring of the quality of EmONC services is crucial for improving them and holding governments to account for providing lifesaving care to all women and newborns in need. The Monitoring Emergency Obstetric Care: A handbook (EmONC handbook) provides comprehensive guidance on how countries can identify service gaps, monitor program implementation, and measure progress [6]. However, the current version contains only three indicators that serve as proxies for quality of care: the intrapartum stillbirth and early neonatal death rate and the direct obstetric case fatality rate. The EmONC handbook is undergoing an extensive revision to reflect advancements in the evidence base on effective interventions, changes in epidemiological patterns, and new approaches to assessing health system functionality (**Box 1**). The revised handbook will also include more focus on newborns, the maternal-infant dyad, and quality of care.

Box 1 **Summary of the Revisioning EmONC project**

The Re-Visioning Emergency Obstetric and Newborn Care (EmONC) project is led by a steering committee coordinated by the Averting Maternal Death and Disability (AMDD) program at Columbia University Mailman School of Public Health and includes the London School of Hygiene and Tropical Medicine, United Nations Children’s Fund, United Nations Population Fund, and the World Health Organization. A broad group of global stakeholder organisations and individuals form the wider technical working group that provides substantive and strategic input throughout the process. The substantive work of review and revision is being conducted through four workstreams and a set of country studies. The entire project is framed and implemented using principles of human-centred design to ensure that the revised EmONC framework meets the needs and real-world conditions at the national and sub-national policy levels and at the frontlines of health systems in low- and middle-income countries at different stages of the obstetric transition. The four workstreams cover the topics of signal functions, levels of care, quality of care, and reviews of country experiences using the existing EmONC indicators and targets.

As part of the Revisioning EmONC project, we conducted a scoping review of the quality of care indicators for emergency obstetric and newborn care with a focus on obstetric emergencies related to direct causes of maternal and neonatal death. This review was followed by a consultative process with technical and programming experts in maternal and newborn health and a prioritisation exercise to select a short list of quality of care indicators for potential inclusion in the EmONC handbook.

## METHODS

We conducted a scoping review, mapping, and prioritisation exercise to identify, assess, and select a short list of quality of care indicators on facility-based emergency obstetric and newborn care. A comprehensive description of the methodology is available in the **Online Supplementary Document**.

**Figure 1:**
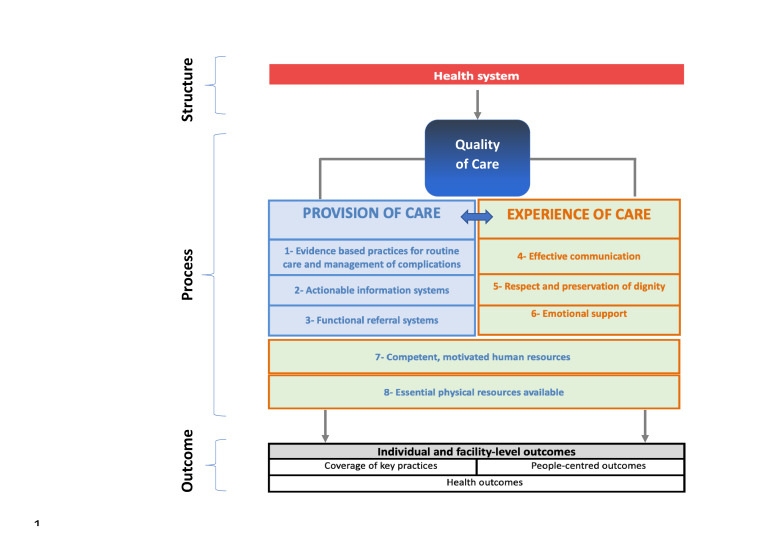
World Health Organization Quality of Care Framework for Maternal and Newborn Health [7]. Licensed under Creative Commons Attribution IGO License.

Our study was guided by the World Health Organization Standards for Improving Quality of Maternal and Newborn Care in Health Facilities (WHO standards document). **Figure 1** presents the framework in the WHO standards document showing how structural and process elements impact facility and individual-level outcomes [7]. Accompanying this framework are a set of standards, quality statements, and associated quality measures.

We focused on the process component of the framework, consisting of provision of care and experience of care domains. The structure component was addressed through other workstreams of the Revisioning EmONC project that focused on the organisation of EmONC services. Therefore, all indicators extracted through the scoping review related to structure (e.g. all input types, including health information systems, policies, financing, medicines and equipment, and human resources indicators) were considered beyond the scope of our study and excluded.

We excluded all indicators related to the outcome component of the framework for two reasons. Although monitoring health outcomes (e.g. maternal mortality, stillbirths, neonatal mortality) is important for assessing the quality of care provided, quality of care is only one of many factors that contribute to individual and facility-based outcomes. A separate consensus-based process is planned for selecting a limited number of outcome measures for inclusion in the revised EmONC handbook.

### Scoping review

We assessed the literature post-publication of the WHO standards document in 2016 [4] to identify any measurement advancements or topical areas missing from it that should be considered for the short indicator list. We identified resources, including peer-reviewed articles and grey literature, through three mechanisms: a review of systematic and scoping reviews on the provision and experience of care, a systematic review of recent primary literature on maternal and newborn experience of care, and resources provided by experts in the Revisioning EmONC project steering committee and quality of care working group. **Table 1** describes the inclusion and exclusion criteria used to select resources for our study.

**Table 1:** Inclusion and exclusion criteria for resources identified through the three mechanisms of the scoping review

Inclusion criteria	For all resources: English language.
	For a review of reviews and expert-recommended resources: At least one indicator related to facility-based maternal or newborn care during the maternal continuum of care (including antenatal care, labour and delivery care, and postnatal care up to 48 h after childbirth). Developed for or relevant to low- or middle-income settings for the provision of care indicators. No geographic constraint for experience of care indicators.
	For systematic review: At least one indicator related to maternal or newborn experience of care. No geographic constraint.
**Exclusion criteria**	Pertaining to quality of care in the community or household.

### Review of reviews for provision and experience of care

We conducted a review of published systematic and scoping reviews on the provision and experience of maternal and newborn care published between 1 January 2016 and 30 March 2021. The start date of the search corresponds with the publication date of the WHO standards document [4]. The WHO standards document was developed based on an extensive literature review and consensus-based process, so we opted to build on this work rather than duplicate it. The WHO standards document was included as a key resource in our study and guided our overall approach to the systematic review and indicator prioritisation process. We searched PubMed, CINAHL and EMBASE. The search strategy is available in Section 2A in the **Online Supplementary Document**.

### Systematic review for experience of care

Because measurement of experience of care is a newer area of research than provision of care, we conducted a systematic review of primary literature on maternal and newborn experience of care published between 1 January 2019 and 17 May 2021. Our start date corresponds with the end date of the search from the most recent systematic review on experience of care published by Larson et al. [8]. We modelled our search strategy on the Larson strategy but modified it to include newborn search terms and had it refined by a Columbia University librarian. We also reviewed all instruments/tools developed to measure maternal experience of care published after 31 October 2017, corresponding with the end date of the search period for the Afulani rapid review on the same topic [9]. We searched PubMed, CINAHL, and EMBASE databases. The full search strategy is available in Section 2B in the **Online Supplementary Document**. Both the Afulani [9] and Larson [8] papers were included in the review of reviews.

### Resources from the grey literature

We solicited resources from the Revisioning EmONC project quality of care workstream and steering committee members. The members of these two groups represent a diverse array of technical and programming experts in the field of maternal and newborn health (Table S16 and Table S17 in the **Online Supplementary Document**). Grey literature sources recommended by these experts included resources from World Health Organization (WHO), Strategies Towards Ending Preventable Maternal Mortality (EPMM), Every Newborn Action Plan (ENAP), the Quality, Equity, Dignity (QED) Network Monitoring Framework, and recommendations submitted by the maternal and newborn health communities in 2021 as part of the revision process of the Service Provision Assessment (SPA) tool [4,5,10,11].

### Indicator extraction and mapping

We extracted indicators from included resources and organised them into an Excel file using the WHO framework categories of structure, process, and outcome. Indicators grouped into the outcome and structure categories, including all indicators labelled as input or outcome type in the WHO standards document, were excluded as described above. Indicators specific to the provision of antenatal care (ANC) were also excluded as out of scope because our study focused on the intrapartum period. A separate Revisioning EmONC project workstream is developing routine signal functions for maternal and newborn care that cover the antenatal and intrapartum periods.

As a second step, the compiled process indicators, encompassing provision of care and experience of care indicators, were organised by relevant standards and associated quality statements in the WHO standards document (Table S8 and S9 in the **Online Supplementary Document**). New categories were created for process indicators that could not be categorised under any quality standard or statement. The purpose of this step was to group similar indicators, enable the removal of duplicates, and facilitate the identification of gap areas (Section 5 in the **Online Supplementary Document**).

Out of the eight WHO standards, standards one and three concern the provision of care (Table S3 in the **Online Supplementary Document**). Of the 16 associated quality statements, two – quality statement 1.1b “mothers and newborns receive routine postnatal care” and quality statement 1.8 “all women and newborns receive care that includes standard precautions for preventing hospital-acquired infections” – were determined to be out of scope (1.1b was considered relevant for the Revisioning EmONC project workstream on routine signal functions, and 1.8 for the workstreams on health system functionality). Indicators grouped under these two statements were excluded. Standards four, five and six are related to experience of care (Table S3 in the **Online Supplementary Document**). All seven associated quality statements were considered within the study scope.

The third step involved reviewing the grouped provision and experience of care indicators and excluding exact duplicates and any remaining indicators deemed out of scope (e.g. indicators on home or community-based care, or input, outcome, ANC-related indicators that had not been removed in earlier steps). For conceptually similar indicators, we selected the indicator that most comprehensively captured the underlying construct of interest. We selected one indicator for each construct where possible, but in some cases, we kept multiple indicators that captured substantively different construct elements (**Table 2**).

**Table 2:** Example of a selected indicator out of a set of duplicate or overlapping indicators

Standard 1: Every woman and newborn receive routine, evidence-based care and management of complications during labour, childbirth and the early postnatal period, according to WHO guidelines.	
**Quality Statement 1.1b: Newborns receive routine care immediately after birth**	
**Indicator**	**Data source**
1. The proportion of all newborns who received all four elements of essential newborn care: immediate and thorough drying, immediate skin-to-skin contact, delayed cord clamping and initiation of breastfeeding in the first hour.	WHO standards document [4]
2. % of newborns who received essential early newborn care (drying, skin-to-skin, delayed cord clamping, breastfeeding).	Quality, Equity, Dignity Indicator Catalogue [5]
3. % receiving immediate care after birth for mother and newborn meeting minimal standard: immediate and thorough drying and immediate skin-to-skin; prophylactic uterotonic; delayed cord clamping; put to breast in first hour after birth.	Recommendation submitted for the SPA revision process [correspondence from maternal newborn health working group]
4. Newborns receiving essential newborn care (%).	Moller 2018 [12]
5. Proportion of newborns who received all four elements of essential care	Das 2018 [13]
6. Newborns receiving essential newborn care – Percentage of newborns who received all four elements of essential newborn care: immediate and thorough drying, immediate skin-to-skin contact, delayed cord clamping and initiation of breastfeeding in the first hour	Benova 2019 [14
7. Proportion of newborns born in the health facility who received early essential newborn care (immediate and thorough drying, immediate skin-to-skin contact, delayed cord clamping and initiation of breastfeeding in the first hour).	Recommendation submitted for the SPA revision process [correspondence from maternal newborn health working group]
**Indicator 1 was selected**	

SPA – Service Provision Assessment Survey; WHO – World Health Organization

### Indicator prioritisation

A consultative process with the Revisioning EmONC project quality of care workstream and steering committee members was conducted to select a short list of indicators from the extracted provision and experience of care indicators. To ensure consistency with other newborn measurement efforts, experts from ENAP were also consulted. A set of criteria was developed and applied to help with the identification of the short list. Table S13 in the **Online Supplementary Document** includes information about the type, geographic location, and year published for the reference associated with each prioritised indicator. To develop the indicator selection criteria, we first compiled criteria used by other similar initiatives and research efforts (Section 4 in the **Online Supplementary Document**). We then prepared an abbreviated list of criteria that we considered most salient – relevant/important, actionable, feasible, evidence-based – and presented it to the quality of care workstream and steering committee members. A consensus was reached to limit the criteria to relevant/important and feasible because all the extracted indicators were evidence-based, and actionability was considered redundant with feasibility (**Table 3**). Agreement was reached that any indicators with evidence of invalidity would be excluded. Although the same indicator selection criteria were applied, separate processes were used to identify the priority provision and experience of care indicators because of differences in how these two topics are commonly measured. Experience of care is typically measured using a scale and the construction of a score based on interviewee responses. Provision of care indicators, in contrast, capture objective information on activities and outputs and are often measured through observations and routine health information systems (e.g. facility registers, patient care records).

**Table 3:** Indicator selection criteria definitions

Selection criteria	Definition
Relevant/important	The indicator is reflected in global guidance, is evidence-based, relates to a leading cause of maternal and newborn deaths or severe morbidity [15,16], and is relevant to emergency obstetric care.*
Feasible	The indicator is feasible to collect with minimum resources and can be acted upon (e.g. it is being collected in health facility tools such as the Service Provision Assessment, Service Availability and Readiness Assessment, Harmonized Health Facility Assessment, or in household or other surveys, or is feasible for collection through routine health information systems).

*Relevance to emergency obstetric care was not an emphasis for selecting experience of care indicators but was central for selecting the provision of care indicators.

#### Provision of care

Based on guidance from the quality of care workstream, the study team selected proxy indicators for each WHO quality statement related to the provision of care. Where appropriate, we combined unique elements from different indicators to develop proxy indicators (**Table 4**). Consensus was reached with the workstream members to select provision of care indicators relevant to complication management and that capture appropriate care, as opposed to harmful practices, so they would all have the same measurement directionality. To assess the feasibility of measuring the prioritised provision of care indicators, we examined available metadata that includes potential data sources for them in the QED catalogue [5] and in the Harmonized Health Facility Assessment tool [21]. Specifically, we reviewed information on data sources listed in the metadata and the likelihood of these sources being regularly available in low-resource settings. We also reviewed measurement guidance submitted by the maternal and newborn health communities to the SPA team during the 2020-2022 SPA revision process (correspondence from maternal newborn health working group).

**Table 4:** Example of developing a proxy indicator for combining elements of indicators that capture parts of the underlying construct of interest

Standard 1: Every woman and newborn receive routine, evidence-based care and management of complications during labour, childbirth and the early postnatal period, according to WHO guidelines.	
**Quality statement 1.4: Women whose progress in labour is delayed or whose labour is obstructed receive appropriate interventions, according to WHO guidelines.**	
**Indicator**	**Data source**
1. The proportion of all women who gave birth in the health facility who underwent instrumental vaginal birth for delayed second stage of labour.	WHO standards document [4]
2. Number of instrumental deliveries/low risk women in labour.	Lazzaretto 2018 [17]
3. Instrumental extraction: using obstetric forceps or vacuum extractor.	Saturno-Hernandez 2018 [18]
4. Instrumental vaginal deliveries (vacuum/forceps).	Rich 2016 [19]
5. Instrumental vaginal delivery.	Boulkedid 2013 [20]
6. The proportion of all women in the health facility with prolonged and/or obstructed labour who gave birth by caesarean section.	WHO standards document [4]
**Indicators 1, 3 and 6 combined to create the following proxy indicator**	
% of women with delayed second stage of labour who underwent instrumental vaginal birth or c-section (disaggregated by mode of delivery).	WHO standards document [4]

WHO – World Health Organization

#### Experience of care

After grouping the experience of care indicators by the WHO standards framework, we further organised them according to the Person-Centered Maternity Care (PCMC) scale [22], including the additional items in the USA version [23]. The PCMC scale has been validated for use in low- and middle-income countries [24-28], and its three sub-scales of communication and autonomy, dignity and respect, and supportive care are consistent with the WHO standards. We matched and compared the extracted experience of care indicators to items in the PCMC scale to assess whether the PCMC items needed updating. For indicators that were not already captured by the PCMC scale, we selected those that met the indicator selection criteria as optional items. Relevance to emergency obstetric care was not emphasised for selecting experience of care indicators, given inconsistent evidence of associations between complications during childbirth and experience of care [29-33]. Further, the study team agreed that every woman, regardless of her complication status, should receive respectful and responsive care.

## RESULTS

**Figure 2:**
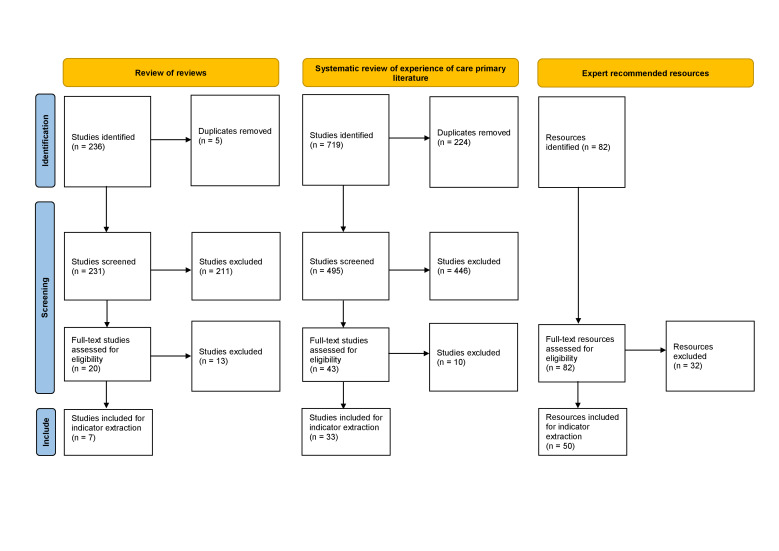
Flow diagram for the scoping review.

**Figure 3:**
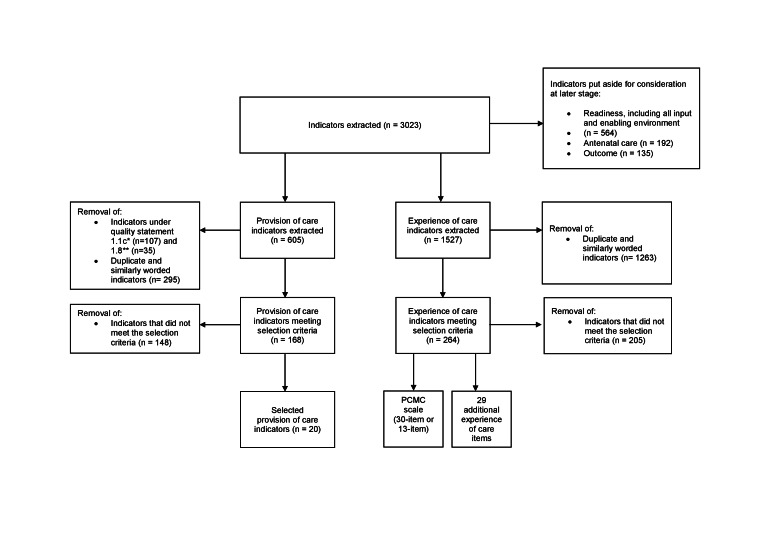
Flow diagram for extracted indicators.

We identified a total of 1037 resources; 90 were included in the study (**Figure 2**). We extracted 3023 indicators from the included resources: 605 provision of care, 1527 experience of care, 564 structure/readiness, 192 ANC, and 135 outcome indicators (**Figure 3**). After excluding the structure, outcome, ANC, and all other out of scope indicators and removing duplicates, the number was reduced to 168 provision of care and 264 experience of care indicators. We were able to group almost all provision of care indicators (92.3%) and experience of care indicators (89.4%) under a WHO standard and associated quality statement (Table S5 and Table S6 in the **Online Supplementary Document**).

After identifying, mapping, and applying the selection criteria to the provision of care indicators, we prioritised 20 for consideration for the revised EmONC handbook (**Table 5** and Table S10 in the **Online Supplementary Document**). Of these, 19 are consistent with indicators in the WHO standards document, although we made minor modifications to 14 of them based on findings from the scoping review (see Table S10 in the **Online Supplementary Document** for notes on any indicator wording deviations from indicators defined in the WHO standards based on updated evidence). We selected one indicator not included in the WHO standards document related to appropriate prophylaxis against disease transmission for women with human immunodeficiency virus (HIV), which will have the most relevance for high prevalence contexts.

**Table 5:** Prioritised provision of care of indicators (total of 20 unique indicators)*

Quality statement	Indicator definition and number	Numerator and denominator
1.1a: Women are assessed routinely on admission and during labour and childbirth and are given timely, appropriate care.	1. % of women who gave birth in the health facility whose blood pressure, pulse, temperature, vaginal examination, and fetal heart sounds were taken during labour, childbirth, and the early postpartum period.†	Numerator: number of women who gave birth in the health facility whose blood pressure, pulse, temperature, vaginal examination, and fetal heart sounds were taken during labour, childbirth, and the early postpartum period (indicator can be disaggregated by each of these stages). Denominator: number of women who gave birth in the health facility.
1.1b: Newborns receive routine care immediately after birth.	2. % of newborns who received all four elements of essential newborn care: immediate and thorough drying, immediate skin-to-skin contact, delayed cord clamping and initiation of breastfeeding in the first hour.†	Numerator: number of newborns who received all four elements of essential newborn care. Denominator: number of newborns.
1.2: Women with pre-eclampsia or eclampsia promptly receive appropriate interventions.	3. % of women with severe pre-eclampsia or eclampsia in the health facility who received magnesium sulphate.†	Numerator: number of women with severe pre-eclampsia or eclampsia who delivered in the health facility and were treated with magnesium sulphate. Denominator: number of women who delivered in the health facility with severe pre-eclampsia or eclampsia.
	4. % of women with severe hypertension in pregnancy in the health facility who received the recommended antihypertensives.	Numerator: number of women with severe hypertension in pregnancy admitted to the health facility who received recommended antihypertensives. Denominator: number of women with severe hypertension in pregnancy who were admitted to the health facility.
1.3: Women with postpartum haemorrhage promptly receive appropriate interventions.	5. % of women with postpartum haemorrhage in the health facility who received therapeutic uterotonic drugs.†	Numerator: number of women with postpartum haemorrhage who delivered in the health facility who received therapeutic uterotonic drugs. Denominator: number of women with postpartum haemorrhage who delivered in the health facility.
	6. % of women in the health facility with postpartum haemorrhage due to a retained placenta for whom manual removal of the placenta was performed.	Numerator: number of women who delivered in the health facility with postpartum haemorrhage due to a retained placenta for whom manual removal of the placenta was performed. Denominator: number of women who delivered in the health facility with postpartum haemorrhage due to a retained placenta.
1.4: Women whose progress in labour is delayed or whose labour is obstructed receive appropriate interventions, according to WHO guidelines.	7. % of women in the health facility with prolonged and/or obstructed labour who gave birth by caesarean section.†	Numerator: number of women with prolonged/obstructed labour who delivered in the health facility who gave birth by caesarean section. Denominator: number of women with prolonged/obstructed labour who delivered in the health facility.
	8. % of women with delayed second stage of labour who underwent instrumental vaginal birth or c-section (disaggregate by mode of delivery).	Numerator: number of women with delayed second stage of labour who gave birth in the health facility who underwent instrumental vaginal birth or c-section (disaggregate by mode of delivery). Denominator: number of women with delayed second stage of labour who delivered in the health facility.
	9. % of women in the health facility with confirmed delay in progress of the first stage of labour who received oxytocin for augmentation.	Numerator: number of women who delivered in the health facility with confirmed delay in progress of the first stage of labour who received oxytocin for augmentation. Denominator: number of women who delivered in the health facility with confirmed delay in progress of the first stage of labour.
1.5: Newborns who are not breathing spontaneously receive appropriate stimulation and resuscitation with a bag-and-mask within one minute of birth, according to WHO guidelines.	10. % of newborns who were not breathing spontaneously who received any positive pressure ventilation using any device (most commonly a bag and mask).†	Numerator: number of newborns (live births and stillbirths excluding macerated stillbirths) who were not breathing spontaneously and who received any positive pressure ventilation using any device (most commonly with bag and mask). Denominator: number of newborns (live births and stillbirths excluding macerated stillbirths) who were not breathing spontaneously.
1.6a: Women in preterm labour receive appropriate interventions for both themselves and their babies, according to WHO guidelines.	11. % of women with preterm pre-labour rupture of membranes who gave birth in the health facility who received prophylactic antibiotics.	Numerator: number of women with preterm pre-labour rupture of membranes who gave birth in the health facility and received prophylactic antibiotics. Denominator: number of women with preterm pre-labour rupture of membranes who gave birth in the health facility.
	12. % of women who gave birth in the facility between 24 and 34 weeks gestational age who received at least one dose of ACS.†	Numerator: number of women who gave birth in the facility between 24 and 34 weeks gestational age and received at least one dose of ACS. Denominator: number of women who gave birth in the facility between 24 and 34 weeks gestational age.
1.6b: Preterm and small babies receive appropriate care, according to WHO guidelines.	13. % of live-born low-birth-weight (<2500 g) newborns born in the health facility who are initiated on KMC or admitted to a KMC unit if a separate unit exists.‡	Numerator: number of admitted low birth weight newborns (<2500 g) who are initiated on KMC anywhere in the facility (disaggregate by <2000 g where possible). Denominator: number of admitted low birth weight newborns (<2500 g) (disaggregate by <2000 g where possible).
1.7a: Women with or at risk for infections during labour, childbirth or the early postnatal period promptly receive appropriate interventions, according to WHO guidelines.	11. % of women who gave birth in the health facility with preterm pre-labour rupture of membranes who received prophylactic antibiotics.§	Numerator: number of women who gave birth in the health facility with preterm pre-labour rupture of membranes who received prophylactic antibiotics. Denominator: number of women who gave birth in the health facility with preterm pre-labour rupture of membranes.
	14. % of women who gave birth in the health facility with signs of infection treated with appropriate antibiotics.†	Numerator: number of women who gave birth in the health facility with signs of infection who received appropriate antibiotics. Denominator: number of women who gave birth in the health facility with signs of infection.
1.7b: Newborns with suspected infection or risk factors for infection are promptly given antibiotic treatment, according to WHO guidelines.	15. % of newborns identified as cases of possible serious bacterial infection in outpatient settings or clinically suspected sepsis in inpatient settings who received at least two days of appropriate injectable antibiotics.†	Numerator: number of newborns identified as cases of possible serious bacterial infection in outpatient settings or clinically suspected sepsis in inpatient settings who received at least two days of appropriate injectable antibiotics. Denominator: number of newborns identified as cases of possible serious bacterial infection in outpatient settings or clinically suspected sepsis in inpatient settings.
3.1: Every woman and newborn is appropriately assessed on admission, during labour and in the early postnatal period to determine whether referral is required, and the decision to refer is made without delay.	16. % of sick, preterm or small newborns who could not be managed at the health facility who were transferred to an appropriate level of care within 1 h of a decision, accompanied by a health care professional and a completed standardised referral note.†	Numerator: number of sick, preterm or small newborns who could not be managed at the health facility who were transferred to an appropriate level of care within 1 h of a decision, accompanied by a health care professional and a completed standardised referral note. Denominator: number of sick, preterm or small newborns who could not be managed at the health facility.
	17. % of pregnant or postnatal women who could not be managed at the health facility who were transferred to a higher-level facility for childbirth or further management without delay, accompanied by a health care professional and a completed standardised referral note.†	Numerator: number of pregnant or postnatal women who could not be managed at the health facility who were transferred to a higher-level facility for childbirth or further management without delay, accompanied by a health care professional and a completed standardised referral note. Denominator: number of pregnant or postnatal women who could not be managed at the health facility.
3.2: For every woman and newborn who requires a referral, the referral follows a pre-established plan that can be implemented without delay at any time.	18. % of pregnant and postnatal women and newborns who were referred with appropriate emergency transport (disaggregate by pregnant woman, postnatal woman, newborn).	Numerator: number of pregnant and postnatal women and newborns who were referred with appropriate emergency transport (disaggregate by pregnant woman, postnatal woman, newborn). Denominator: number of pregnant and postnatal women and newborns who were referred (disaggregate by pregnant woman, postnatal woman, newborn).
3.3: For every woman and newborn referred within or between health facilities, there is appropriate information exchange and feedback to relevant health care staff.	19. % of referred women and newborns seen at the referring facility who received timely care at the referral facility (disaggregate by women, newborns).	Numerator: number of referred women and newborns seen at the referring facility who received timely care at the referral facility (disaggregate by women, newborns). Denominator: number of referred women and newborns seen at the referring facility (disaggregate by women, newborns).
Not listed in the WHO standards document	20. % of women living with HIV who delivered in the health facility and received appropriate prophylaxis to prevent mother-to-child transmission and antiretroviral therapy.	Numerator: number of women living with HIV who delivered in the health facility who received appropriate prophylaxis to prevent mother-to-child transmission and antiretroviral therapy. Denominator: number of women living with HIV who delivered in the health facility.

ACS – antenatal corticosteroids, KMC – kangaroo mother care, HIV – human immunodeficiency virus, WHO – World Health Organization

*We considered core references as the World Health Organization’s Standards for Improving Quality of Maternal and Newborn Care in Health Facilities; Quality, Equity, Dignity catalogue; and Every Newborn Action Plan metric recommendations. All indicators should be measured based on a specified time period.

†Indicators identified as collectable through routine information systems.

‡Kangaroo mother care is defined as care of preterm infants carried skin-to-skin with the mother. Key features include continuous skin-to-skin contact with the mother, exclusive breastfeeding, and early discharge from the hospital in the kangaroo position with frequent home visits by health workers [10].

§Duplicate indicator that was categorised under two World Health Organization’s Standards.

Consensus was reached through our consultation processes that experience of care is best measured using a composite score that captures different aspects of a person’s experience. We, therefore, selected the PCMC scale for inclusion in the revised EmONC handbook because it is the most comprehensive scale for measuring women’s experiences during childbirth. Each question on the scale (**Table 6** has response options ranging from zero to three that have been psychometrically tested for generating a composite score. Adding up the responses to the individual items and rescaling the totals (using procedures that would be described in the revised handbook if the PCMC scale is added) will generate a score ranging from zero to 100, where higher scores represent more positive experiences. Although the USA version of the PCMC scale has more items, we selected the 30-item version because it has been validated in settings where the EmONC handbook will most likely be used. Where the 30-item version is not practical, we recommend the shorter 13-item version, which has also been psychometrically validated [26]. The 13-item version, however, excludes the items on birth companionship and verbal and physical abuse, which are critical issues that can be remedied through health system reforms. Thus, we recommend that these three items be included in EmONC assessments or other health facility surveys as additional items if the shorter PCMC scale is used.

**Table 6:** Person-Centered Maternity Care scale

Person-Centered Maternity Care 30-item scale*	Included in the 13-item scale (yes or no)
**Supportive care**	
1. How did you feel about the amount of time you waited?	No
2. Were you allowed to have someone you wanted to stay with you during labour?	No
3. Were you allowed to have someone you wanted to stay with you during the delivery?	No
4. When you needed help, did you feel the doctors, nurses, midwives, or other staff at the facility paid attention?	Yes
5. Did the doctors, nurses, midwives, or other staff at the facility talk to you about how you were feeling?	Yes
6. Did the doctors, nurses, midwives, or other staff at the facility support your anxieties and fears?	No
7. Do you feel the doctors, nurses, midwives or other staff did everything they could to help control your pain?	No
8. Did you feel the doctors, nurses, midwives, or other staff at the facility took the best care of you?	Yes
9. Did you feel you could completely trust the doctors, nurses, midwives, or other staff at the facility with regards to your care?	No
10. Thinking about the wards, washrooms, and the general environment of the health facility, will you say the facility was very clean, clean, dirty, or very dirty?	No
11. Do you think there was enough health staff in the facility to care for you?	No
12. Thinking about the labour and postnatal wards, did you feel the health facility was crowded? (Revised wording: did you feel the place you gave birth was crowded during your birth stay (e.g. not enough beds, moved from room to room, being in triage a long time?))	No
13. In general, did you feel safe in the health facility?	No
14. Was there water in the facility?	No
15. Was there electricity in the facility?	No
**Dignity and respect**	
16. Did the doctors, nurses, midwives, or other staff at the facility treat you with respect?	Yes
17. Did the doctors, nurses, midwives, and other staff at the facility treat you in a friendly manner?	Yes
18. During examinations in the labour room, were you covered up with a cloth or blanket or screened with a curtain so that you did not feel exposed?	Yes
19. Do you feel like your health information was or will be kept confidential at this facility?	No
20. Did you feel the doctors, nurses, midwives, or other health providers shouted at you, scolded, insulted, threatened, or talked to you rudely?	No
21. Did you feel like you were treated roughly like pushed, beaten, slapped, pinched, physically restrained, or gagged?	No
**Communication and autonomy**	
22. During your time in the health facility, did the doctors, nurses, midwives, or other health care providers introduce themselves to you when they first came to see you?	No
23. Did the doctors, nurses, midwives, or other health care providers call you by your preferred name?	Yes
24. Did you feel like the doctors, nurses, midwives, or other staff at the facility involved you in decisions about your care?	Yes
25. Did the doctors, nurses, midwives or other staff explain to you why they were doing examinations or procedures on you?	Yes
26. Did the doctors, nurses, midwives or other staff explain to you why they were giving you any medicine?	Yes
27. Did the doctors, nurses, midwives, or other staff at the facility ask your permission/consent before doing procedures and examinations on you?	Yes
28. During the delivery, do you feel like you were able to be in the position of your choice?	Yes
29. Did the doctors, nurses, midwives, or other staff at the facility speak to you in a language or in terms you could understand?	No
30. Did you feel you could ask the doctors, nurses, midwives, or other staff at the facility any questions you had?	Yes

*Items from the 30-item Person-Centered Maternity Care scale are measured on a 4-point scale from zero to three (e.g. 0 – no, never; 1 – yes, a few times; 2 – yes, most of the time; 3 – yes, all the time).

We selected an additional 29 experience of care items that cover areas not captured by the PCMC scale, including newborn experience of care, continuity of care, physical accessibility, issues around payment for services (or lack thereof), family-centred care, and bereavement care (**Table 7**). Many newborn items and items concerning mother-baby separation require additional research to ensure valid measurement.

**Table 7:** Additional 29 experience of care items, organised by mother and newborn

Additional experience of care items	Source
**Mother**	
1. The proportion of all women discharged from the labour and childbirth area of the facility who reported receiving written and verbal information and counselling on the following elements before discharge: nutrition and hygiene, birth spacing and family planning, exclusive breastfeeding and maintaining lactation, keeping their baby warm and clean, communication and play with the baby, danger signs for the mother and newborn and where to go in case of complications.	WHO standards document [4]
2. The proportion of all women who gave birth in the health facility who reported that they were given the opportunity to discuss their concerns and preferences.	WHO standards document [4]
3. The proportion of women who reported that they were told different things by different care providers about their health that led to confusion.	Wong 2013 [34]
4. The proportion of all women who gave birth in the health facility who reported that health care staff showed good knowledge of their history and the care that had been given to date.	WHO standards document [4]
5. The proportion of women who reported that health providers sexually harassed them or made sexual advances (for example, inappropriate touching or sexual comments that make them feel uncomfortable).	Freedman 2018 [35]
6. The proportion of mothers who reported not being cleaned after birth and third stage of labour.	Banks 2018 [36]
7. The proportion of women who reported being instructed to clean up blood, urine, faeces or amniotic fluid.	Bohren 2018 [37]
8. The proportion of women who reported being denied care for any reason.	Afulani 2020 [9]
9. The proportion of women who reported being detained at facilities due to lack of payment.	Afulani 2020 [9]
10. The proportion of women who gave birth in the health facility who reported being aware of the existence and location of a complaints box.	WHO standards document [4]
11. The proportion of all women in the health facility who made a complaint and whose complaints were acted upon without repercussions.	WHO standards document [4]
12. The proportion of procedures in the health facility that require written consent for which there is an associated record of consent signed by the woman or a family member.	WHO standards document [4]
13. The proportion of carers in the health facility who report having received information about the care plan for their newborn.	Recommendation submitted for the SPA revision process [correspondence from maternal newborn health working group]
14. The proportion of parents who reported feeling that the staff at the local referring hospital explained the reason for the transfer of their baby (only applies if the baby was transferred from another unit).	Thyagarajan 2018 [38]
15. The proportion of mothers (carers) who reported being supported in family-cantered care (facility allows companion/family member, there is a place for a family member to sleep, place for a family member to eat, place for a family member to bathe).	Recommendation submitted for the SPA revision process [correspondence from maternal newborn health working group]
16. The proportion of women who reported having direct access to the bathroom in the room they were lying after giving birth.	Baranowska 2020 [39]
17. The proportion of women with disabilities who reported that the hospital, clinic, or health care provider(s) office was accessible given their needs (e.g. specialised equipment, extra space).	Wong 2013 [34]
18. The proportion of all women undergoing bereavement or an adverse outcome who reported receiving additional emotional support from health facility staff.	WHO standards document [4]
19. The proportion of women who reported being discharged too early after birth.	Ziabakhsh 2018 [40]
20. The proportion of women who reported being separated from their baby without medical indication.	Azhar 2018 [41]
21. The proportion of women who reported being encouraged and/or able to mobilise during labour.	Bohren 2018 [37]
22. The proportion of all healthy mothers on postnatal wards or areas in the health facility who reported receiving breastfeeding counselling and support from a skilled health care provider.	WHO standards document [4]
23. The proportion of women who reported being asked for bribes or payments other than the official payment.	Afulani 2018 [24]
24. The proportion of women who reported that they were treated differently because of any personal attribute such as their age, marital status, number of children, education, wealth, sexual orientation, race/ethnicity/tribe, connections with the facility, etc.	Afulani 2022 [23]
**Newborn**	
25. The proportion of women who reported feeling that their newborn’s health information was or would be kept confidential at the facility.	Adapted from WHO standards document [4]
26. The proportion of women who reported their newborns were maltreated.	Recommendation submitted for the SPA revision process [correspondence from maternal health working group]
27. The proportion of newborns who had prompt removal of soiled wrapper or diaper and cleaning of urine and faeces.	Sacks 2017 [42]
28. The proportion of carers in the health facility who report having received information about the care plan for their newborn.	Recommendation submitted for the SPA revision process [correspondence from maternal health working group]
29. The proportion of carers of small and sick newborns who reported receiving appropriate developmental supportive care for the newborn during their stay in the health facility.	Recommendation submitted for the SPA revision process [correspondence from maternal health working group]

SPA – Service Provision Assessment survey, WHO – World Health Organization

### Feasibility

A review of the recommendations submitted for the 2020-2022 SPA revision process (Tables S14 and S15 in the **Online Supplementary Document**) showed that two of our selected provision of care indicators (related to essential newborn care and neonatal resuscitation – indicators two and ten in **Table 5**) were recommended for inclusion in the SPA as core indicators. Indicators for vital sign assessment for women, fetal heart measurement, administration of uterotonic drugs, and prevention of maternal-to-child transmission were recommended for inclusion in an optional module. Regarding experience of care, the 13-item PCMC scale was recommended for retention. After consulting the QED catalogue, we determined that potentially, 12 out of our recommended 20 provision of care indicators can currently be collected through routine health information systems (Table S14 in the **Online Supplementary Document**). The other eight indicators can be collected through surveys and special studies.

### Gap areas

After organising the compiled provision and experience of care indicators by the relevant WHO standards and associated quality statements, provider experience and patient safety emerged as gap areas. Although components of patient safety and provider experience are addressed in the WHO standards document through quality statements 1.9, 5.2, and 4.2, respectively, our review identified few indicators on these topics. We conducted rapid literature searches for both patient safety and provider experience because we recognised that our scoping review search criteria may have resulted in the inadvertent exclusion of indicators on these topics (Section 5 in the **Online Supplementary Document**). The literature searches yielded no additional patient safety measures and few health provider experience measures beyond burnout. High levels of provider burnout during the coronavirus disease 2019 (COVID-19) pandemic have elevated the issue of provider experience [43-46], yet consensus has not been reached on a comprehensive set of provider experience measures.

## DISCUSSION

From this review, we selected 20 provision of care indicators for potential inclusion in the EmONC handbook and for use in other monitoring efforts. For experience of care, we recommend that the revised handbook includes a score based on the PCMC scale. We also recommend an additional menu of 29 items to guide more in-depth experience of care assessments. Our study was grounded in the WHO standards document [4] and involved consultations with maternal and newborn experts to ensure our recommendations were aligned with existing guidelines and the latest measurement efforts.

One goal of our study was to assess the evidence base to identify any measurement improvements or emergence of new priority areas since the WHO standards document was published in 2016. We found that almost all indicators identified from our scoping review could be categorised under an existing WHO standard and associated quality statement, and 19 of the 20 prioritised provision of care indicators are consistent with those in the WHO standards document. Thus, our study reaffirmed the continued value of the WHO standards document for guiding maternal and newborn quality of care measurement and monitoring efforts.

Around half of the recommended provision of care indicators can potentially be collected through routine health information systems, and the others through available health facility survey platforms or special studies. For the experience of care, we selected an indicator based on an existing scale that has been validated for use in low- and middle-income settings where most EmONC assessments are likely to occur [22,24,26,28,47-50] but also recommended adding additional indicators where possible. These findings suggest that our recommended indicators can be regularly collected in most locations, although implementation research in resource-constrained settings would further confirm measurement feasibility.

Provider experience and patient safety emerged as gap areas from our scoping review and consultation processes. Future research efforts should systematically assess available validated tools for measuring provider experience to inform the development of provider experience indicators. Similarly, more work is needed to develop a set of consensus-based indicators for patient safety that capture the complex issues related to harmful practices such as negligence, overmedicalisation (inappropriate interventions such as unnecessary episiotomy or non-medically indicated caesarean sections), antibiotic resistance, and lack of training on standards of care [51].

Our scoping review approach, which included three mechanisms to identify materials, had some limitations. We only included a select set of databases and English-language articles, which may have resulted in us missing key articles available through other databases and in different languages. Although we modified the Larson search strategy to include newborn as well as maternal tools for the systematic review component, we may have missed newborn tools published before the Larson systematic review since we used the end date of the Larson search as our start date. The search terms used for the review of systematic and scoping reviews constrained our results to only articles that included both maternal and newborn indicators rather than articles that included maternal, newborn, or both maternal and newborn indicators (Section 2A in the **Online Supplementary Document**). We opted not to redo the search because we agreed that the large volume of resources already identified through the three mechanisms was sufficient for our study.

Because our search terms were restricted to maternal and newborn care, indicators around the quality of abortion care, often categorised separately as part of sexual and reproductive health care, were not captured in our scoping review [52]. Additional consultations for the Revisioning EmONC project are being conducted on the quality of abortion care so that this topic is adequately reflected in the revised handbook.

We acknowledge that some of our recommended indicators require additional measurement work, including the indicators on referral, care for small and sick newborns, maternal and newborn separation, and on the newborn experience of care. We recommend that our short list is regularly reviewed and revised to reflect improvements in measurement on these topic areas. Our recommendations are restricted to the provision of care for women and newborns with complications in the intrapartum period and experience of care. This focus is consistent with the recommendations in the Lancet Commission on High Quality Health Systems to include competent care and user experience in assessing the quality of care [3]. Fully evaluating the availability, accessibility, and quality of emergency obstetric and newborn care requires also examining health system functionality and health outcomes. For health care workers to be able to provide high-quality emergency obstetric and newborn care, they need to be embedded in an enabling environment. Similarly, improvements in health outcomes and women’s and newborns’ experiences of care start with women being able to reach health facilities in a timely manner [53].

The findings from this study are inputs to the broader Revisioning EmONC project, which addresses all three components of structure, process, and outcomes as described in the WHO standards document, as well as the continuity of care during pregnancy, labour, delivery and the immediate postpartum period. Health outcome indicators are important for monitoring quality of care, and, as noted, a separate process is planned to select a limited number of outcome measures for inclusion in the revised EmONC handbook. Of note, our recommended indicators are intended for program monitoring. The goal is a short set of indicators that will serve as red flags to prompt action, including additional data collection, to understand the source of poor care better so it can be addressed. Thus, the focus is on parsimony rather than comprehensiveness. The full set of indicators and signal functions selected for the Revisioning EmONC project is expected to undergo wider vetting with the maternal and newborn communities and to be tested using a human-centred design approach in selected countries. Our recommended indicators will be included in these processes for further validation.

## CONCLUSIONS

We identified a short set of evidence-based provision and experience of care indicators that are consistent with global standards, can be measured through existing tools and platforms, and can be used for monitoring and planning purposes in low-resource settings. Their potential inclusion in the revised EmONC handbook and in other monitoring activities could increase the use of data for decision-making on programs and resource allocation and help hold decision-makers to account for improving the quality of emergency obstetric and newborn care and, ultimately, women’s and newborns’ lives.

## Additional material


Online Supplementary Document
