# Supplementary material for: A scoping review, mapping, and prioritisation process for emergency obstetric and neonatal quality of care indicators: Focus on provision and experience of care
Source: J Glob Health. 2023 Oct 13;13:04092. doi: 10.7189/jogh.13.04092 (PMC10569369; doi:10.7189/jogh.13.04092)
Supplement: Online Supplementary Document [file jogh-13-04092-s001.pdf]

Complete Methods

By: WS3 study team (Patience Afulani, Jennifer Requejo, Dee Wang, Emma Sacks, Ntemena Kapula, Jacqueline Odiase, Erica Munson, Alisha Sarakki) on behalf of the WS3 working group

METHODS

Section 1. Study Design

We carried out an indicator mapping exercise (OSF preregistration doi: 10.17605/OSF.IO/MSXBD) to identify and prioritize measures of quality of care as part of a process to revise the EmONC framework. The current EmONC framework includes two indicators related to quality of care – intrapartum stillbirth rate and direct obstetric case fatality rate. These are both health impact measures that are reflective of service availability and readiness. This review is an integral step in determining how to complement these two measures and better integrate quality of care into the EmONC framework. A key objective of the EmONC revision process is to incorporate newborn measures. Hence, our study examined quality of care measures for both mother and newborn.

The mapping exercise used as its starting point the WHO quality of care framework for maternal and newborn health (WHO, 2016). This WHO framework is grounded on Donabedian's conceptualization of quality (Donabedian, 1988) which includes the dimensions of structure (human resources, equipment and supplies, and strong health system including the health information system), process (technical and interpersonal or experiential aspects of care, or "provision of care" and "experience of care", as well as managerial processes), and health and person-centered outcomes. Our work, and the overall EmONC revisioning project, also connects with the concept of effective coverage. The provision of care and experience of care indicators we worked with correspond with the quality adjusted step of the effective coverage cascade that was adapted by the Effective Coverage Think Tank Group (Marsh 2020). While there has been an exponential increase in research on each of Donabedian's dimensions of quality of care since the WHO framework was published, indicators to measure the provision and experience of care for maternal and newborn health (with an emphasis on emergency obstetric care) have not been synthesised together. A core subset of these indicators has also not yet been prioritised for emergency obstetric care planning and monitoring. This study is not intended to be a systematic or comprehensive review, but rather it provides: 1) a scoping of available indicators on provision and experience of maternal and newborn care from key resources, 2) a process for selecting a core set of indicators from this list useful for assessments of emergency obstetric care, and 3) a final indicator set reached through consensus with the quality of care working group and the EmONC steering committee.

The "structure" dimension of the WHO framework overlaps with concepts of health system "readiness" and the "enabling environment" for health care workers, and a more recent formulation of the health system as composed of "hardware" and "software" dimensions. Numerous activities have been conducted or are currently underway around health system readiness indicators for maternal and neonatal care, including an inventory of 600 indicators (Moxon et al., 2020) and a recently completed revision process for the Service Provision Assessment tool (SPA MNH Working Group, 2021) that involved an extensive consultation with the maternal and newborn community. Other service assessment tools or guidance materials include the harmonized health facility tool, the Primary Health Care Performance Initiative scorecards, the primary health care monitoring and evaluation framework,

and tools generated for the NEST 360, ENAP, and EPMM initiatives amongst others. The World Bank is also currently championing work around redesigning health services.

The revised EmONC handbook will build on these activities and existing data collection instruments to provide guidance on assessing readiness. Recommendations on assessing readiness are being developed through a separate exercise led by Columbia University's Averting Maternal Deaths and Disability (AMDD) team.

Section 2. Resource identification and inclusion criteria

Resources for the scoping review were identified in 3 ways.

A) Review of reviews (systematic and scoping).
   - We had intended to do a "review of reviews" to identify systematic and scoping reviews published post 2016 (post publication of the WHO Standards to avoid duplicating this work and to leverage the findings from it) that met our inclusion criteria: studies published in English, provision of care studies based in LMICS or global guidance (experience of care studies were not limited by geographic constraints), facility-based care during the maternal and newborn continuum of care (including antenatal care, intrapartum care, and up to 48 hours postpartum to capture newborn indicators), and one or more indicators related to maternal or newborn quality of care.
   - Search strategy

     ((((maternal) AND (neonatal OR newborn)) AND (quality of care)) AND (indicators))

   - We realized later in the review process that the original search strategy had been too restrictive (requiring maternal AND newborn terms rather than maternal OR newborn terms). In the interest of time, and belief that the resources identified by the previous two steps were sufficiently comprehensive, we did not redo the review of reviews.

B) Systematic review of primary literature on experience of care
   - Because the body of research on experience of care is more recent I and less extensive than the body of research on provision of care, we conducted a systematic review of primary literature specifically on maternal and newborn experience of care.
   - Our "anchor" or baseline documents for this search included a rapid review on experience of care (Afulani et al., 2018; Afulani et al., 2020), and a scoping review of published literature on measures/instruments for maternal experience of care (Larson et al., 2020).
   - Because the search period for the Larson scoping review was January 1, 2007 through January 1, 2019, we selected January 1, 2019 as the start date for our systematic search for studies with maternal and newborn experience of care indicators. The end date was May 17, 2021.
   - We extracted the indicators in the instruments identified by the Larson 2020 scoping review that were published after the end date (October 31, 2017) of the Afulani rapid review (Afulani et al., 2018; Afulani et al., 2020). However, a limitation of this approach was that the Larson 2020 review focused on maternal instruments so we may have missed newborn indicators published between January 1, 2017 (the end date of a review on disrespect and

abuse of newborns (Sacks, 2017) that we reviewed) and January 1, 2019 (the start date of our systematic search).

- Our search strategy was modelled on the Larson 2020 search strategy. Because the Larson review search strategy mostly captured maternal indicators, we added newborn search terms and had the strategy reviewed and refined by a Columbia University librarian. Below is the search strategy for PubMed. The strategy was also modified for CINAHL and EMBASE.

  (maternal health[tiab] OR maternal service*[tiab] OR maternity care[tiab] OR maternal care[tiab] OR maternity service*[tiab] OR "Maternal Health"[mesh] OR "Maternal Health Services"[mesh] OR newborn health[tiab] OR newborn service*[tiab] OR newborn care[tiab] OR "infant, newborn"[mesh] OR "Newborn Health Services"[tiab] OR infant health[tiab] or infant service*[tiab] OR infant care[tiab] OR infant service*[tiab] OR infant health services[tiab] OR neonate health[tiab] OR neonatal health[tiab] OR neonatal service*[tiab] OR neonatal care[tiab] OR neonate care[tiab] OR neonat* service*[tiab] OR neonat* health service*[tiab] AND (experience*[tiab] OR person-centered[tiab] OR patient-centered[tiab] OR woman centered[tiab] OR women centered[tiab] OR newborn centered[tiab] OR neonate centered[tiab] OR client centered[tiab] OR satisfaction[tiab] OR social support*[tiab] OR emotional support*[tiab] OR provider choice[tiab] OR choice of provider[tiab] OR wait time*[tiab] OR affordability[tiab] OR dignity[tiab] OR respect[tiab] OR privacy[tiab] OR confidentiality[tiab] OR discrimination[tiab] OR communication[tiab] OR disrespect[tiab] OR abuse[tiab] OR mistreatment[tiab] OR perception*[tiab] OR legal accountability*[tiab] OR bereavement and posthumous care[tiab])

- Inclusion criteria: studies published in English, facility-based care during the maternal continuum of care (including antenatal care and 48 hours postpartum to capture newborn indicators), and one or more indicators related to maternal or newborn experience of care.

C) Expert recommended resources: an initial list of resources was compiled by the core study group (Quality of care workstream co-chairs and research assistants) and circulated to all members of the Quality of care workstream (workstream 3) of the Revisioning EmONC project for additional resource recommendations.

- Resources included documents and reports from the World Health Organization (WHO), the Ending Preventable Maternal Mortality (EPMM) initiative, Every Newborn Action Plan (ENAP), Quality of Care Network, Lancet Global Health Commission on High Quality Health Systems
- Resources identified also included published articles that were NOT identified via the other two search strategies.
- Inclusion criteria for resources on provision of care indicators: reports, global guidance documents, resources and literature related to provision of care based in LMICs;; facility-based care during the maternal continuum of care (including antenatal care and 48 hours postpartum to capture immediate maternal and newborn health indicators); and containing one or more indicators related to maternal or newborn quality of care.

- Inclusion criteria for resources on experience of care indicators: reports, global guidance documents, resources and literature related to the experience of care (not limited by geographic constraints); facility-based care during the maternal continuum of care (including antenatal care and 48 hours postpartum to capture immediate maternal and newborn health indicators); and containing one or more indicators related to maternal or newborn quality of care.
- Although the Health Systems Strengthening compendium, WHO Service Availability and Readiness Assessment (SARA), Service Provision Assessment (SPA), and WHO Surgical Assessment Tool (SAT) were identified as key documents by Workstream members, indicators were not extracted from them for the purposes of this scoping review, which was limited to provision and experience of care indicators. These documents were instead used as references for the separate process led by AMDD (noted above) on developing guidance for assessing facility readiness to deliver emergency obstetric care.

Figure S1. WHO Standards Framework

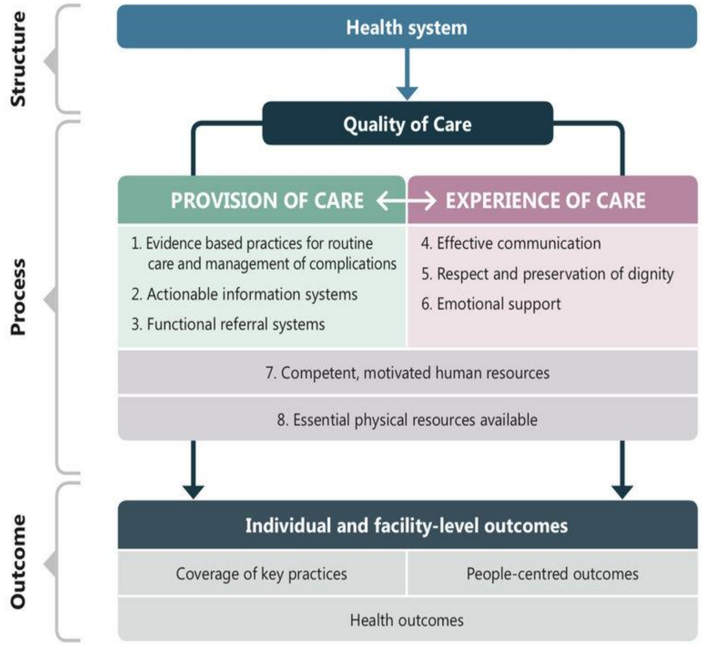

Note: Figure S1 from WHO Standards for improving quality of maternal and newborn care in health facilities (WHO, 2016) and and Tunçalp Ö., Were W, MacLennan C, Oladapo O, Gülmezoglu A, Bahl R, et al. Quality of care for pregnant women and newborns—the WHO vision. BJOG Int J Obstet Gynaecol. 2015;122:1045–9. doi:10.1111/1471-0528.13451.

Table S1. Summary of resources by category for scoping review

	Dimension of care	Time frame	Number of articles for screening	Number of articles for full text review	Number for indicator extraction
A) Review of reviews (systematic and scoping)	Provision of care and experience of care	2016-Mar 2021	231	20	7
B) Systematic review of primary literature	Experience of care	Jan 2019 – May 2021	495	43	33
C) Expert recommended resources	Provision of care, experience of care	N/A	N/A	82	50

Section 3. Extraction and mapping

In total, we extracted 3023 indicators: 605 provision of care, 1527 experience of care, 564 structure/readiness indicators, 192 ANC and 135 outcome indicators. The provision of care, experience of care, and readiness indicators were each compiled into separate excel files. The verbatim indicator and its source document were recorded. A set of sequential and consultative steps were followed to reach consensus on a core set of provision and experience of care indicators. The readiness indicators were shared with the AMDD team as previously described.

Steps 1 and 2 involved organizing the indicators according to the 3-level structure outlined in the WHO quality of care framework – 8 standards, one for each WHO quality of care framework domain; 2-3 quality statements per standard; set of quality measures (input, output, and outcomes) for each statement (Table S2) (WHO, 2016).

Step 1. Categorizing indicators by indicator type – input, output/process, outcome

All indicators were then categorized by indicator type based on the definitions in the WHO standards document (input, output/process, and outcome) (Table S4). According to these definitions, **input** measures include “physical resources, policies, protocols and guidelines, **output** measures include coverage of key practices that are mostly expressed as proportions, and **outcome** measures “include people centred and health outcomes” (Table S2, quality measures description).

All indicators categorized as input measures were removed from the provision and experience of care excel files and grouped with the facility readiness indicators. Cross-cutting indicators on facility readiness such as human and physical resources, WASH, information systems, and inputs (financial,

policy) were also placed in a separate file and shared with the AMDD team to help inform their work on health system readiness. We truncated the extraction of the cross-cutting indicators to avoid duplicating other efforts and to leverage existing work on readiness. Antenatal care (ANC) indicators were placed into a separate file due to uncertainty on whether these indicators were in scope and would ultimately be included as part of a core set of quality measures for the EmONC handbook. These indicators will help inform a separate research step led by the AMDD team to develop routine signal functions for the handbook, which will likely include the antenatal period. Additionally, all outcome measures including maternal mortality and morbidity, direct obstetric case fatality rate, newborn mortality and morbidity and stillbirth prevalence indicators were moved to a separate excel file for health impact indicators. Agreement on the salient health impact/outcome indicators to include in the EmONC framework should be reached through consensus in the EmONC steering committee.

Step 2: Grouping by WHO Quality of Care Standards (8 total) and Quality of Care Statements (31 total)

Categorizing of the 8 quality of care standards showed that standards 1 and 3 are related to provision of care, 4, 5 and 6 are linked to experience of care, and 2, 7 and 8 are related to readiness. All indicators grouped under standards 2, 7, and 8 were placed in the excel file on readiness and shared with the AMDD team.

We organized the indicators into the provision of care and experience of care dimensions according to the 8 standards of care (Table S3) and their associated 31 quality statements (WHO, 2016). The purpose of this step was to: 1) facilitate the grouping of similar indicators together under the relevant quality standard and associated quality statement, 2) determine whether to retain all 8 quality standards and related indicators, 3) enable the removal of duplicates, and 4) enable identification of gap areas.

Indicators that could not readily be organized under the quality standards or statements related to provision and experience of care were placed in an “uncategorized” group. Once indicators had been organized under the quality standards or statements, they were further categorized under the associated quality measures. We created new categories (61 for provision of care and 121 for experience of care) for indicators that could not be categorized under a WHO Standard quality measure. For example, if there were indicators around bathing newborns, a “bathing” category was created under the quality statement 1.1b “newborns receive routine care immediately after birth”. There were also new categories created for indicators that did **not** fall under a WHO **standard** or **quality statement** (e.g. Indicators around contraception prevalence).

We screened the Quality Statements related to Provision and Experience of Care for importance and relevance. Of the 16 Quality Statements related to provision of care, two (Quality Statement 1.1b mothers and newborns receive routine postnatal care and Quality Statement 1.8 all women and newborns receive care that includes standard precautions for preventing hospital-acquired infections) were determined to be out of scope for our study, and indicators associated with these two statements were removed. These two statements are relevant for defining a set of routine signal functions and this information was shared with the AMDD team working on that research step. Of the 7 quality statements related to experience of care, the study team agreed that all were salient for the EmONC handbook revision project.

Table S2. Definitions from the WHO Standards for improving quality of maternal and newborn care in health facilities

Term	Definition
Standard	"A description of what is expected to be provided to achieve high-quality care around the time of childbirth"
Quality Statement	"Concise statements of priorities for measurably improving quality of care around childbirth. They define the markers of quality derived from evidence on the thematic area and the resources required"
Quality Measures	"Criteria for assessing, measuring and monitoring the quality of care as specified in the quality statement. They are of three types:<br>**Input**: what must be in place for the desired care to be provided (e.g. physical resources, human resources, policies, guidelines)<br>**Output**: whether the desired process of care was provided as expected<br>**Outcome**: the effect of the provision and experience of care on health and people-centred outcomes"

Table S3. Standards mapped to the categories of Provision of Care, Experience of Care and Readiness*

Dimension	Standards
Provision of care	Standard 1: Every woman and newborn receive routine, evidence-based care and management of complications during labour, childbirth and the early postnatal period, according to WHO guidelines.
	Standard 3: Every woman and newborn with condition(s) that cannot be dealt with effectively with the available resources is appropriately referred.
Experience of care	Standard 4: Communication with women and their families is effective and responds to their needs and preferences.
	Standard 5: Women and newborns receive care with respect and preservation of their dignity.
	Standard 6: Every woman and her family are provided with emotional support that is sensitive to their needs and strengthens the woman's capability.
Readiness (including information systems and water, sanitation, hygiene and health)	Standard 2: The health information system enables use of data to ensure early, appropriate action to improve the care of every woman and newborn.
	Standard 7: For every woman and newborn, competent, motivated staff are consistently available to provide routine care and manage complications.
	Standard 8: The health facility has an appropriate physical environment, with adequate water, sanitation and energy supplies, medicines, supplies and equipment for routine maternal and newborn care and management of complications.

*Note: Input type indicators that are related to readiness were included in standards 1-6. Table S4 shows the breakdown of indicator type. All input measures were grouped into the readiness category, mapped onto the associated quality standards, quality statements and measures, and shared as a separate file with the AMDD team.

Table S4. Summary of number of input, output/process, outcome indicators from the WHO Standards for improving quality of maternal and newborn care in health facilities by dimension, standard and quality statement

	Input	Output/process	Outcome	Total
**STANDARD 1 (provision of care)**	**57**	**56**	**42**	**155**
Quality statement 1.1a	4	5	3	12
Quality statement 1.1b	6	5	1	12
Quality statement 1.1c	8	9	2	19
Quality statement 1.2	3	3	3	9
Quality statement 1.3	4	2	5	11
Quality statement 1.4	5	7	4	16
Quality statement 1.5	5	2	2	9
Quality statement 1.6a	4	3	3	10
Quality statement 1.6b	4	3	4	11
Quality statement 1.7a	3	5	2	10
Quality statement 1.7b	4	2	3	9
Quality statement 1.8	7	4	3	14
Quality statement 1.9	0	6	7	13
**STANDARD 3 (provision of care)**	**9**	**10**	**4**	**23**
Quality statement 3.1	3	3	1	7
Quality statement 3.2	3	4	3	10
Quality statement 3.3	3	3	0	6
**PROVISION OF CARE TOTALS**	**66**	**66**	**46**	**178**
**STANDARD 4 (experience of care)**	**8**	**5**	**6**	**19**
Quality statement 4.1	4	3	4	11
Quality statement 4.2	4	2	2	8
**STANDARD 5 (experience of care)**	**15**	**10**	**9**	**34**
Quality statement 5.1	3	2	3	8
Quality statement 5.2	8	5	3	16
Quality statement 5.3	4	3	3	10
**STANDARD 6 (experience of care)**	**9**	**6**	**5**	**20**
Quality statement 6.1	4	2	1	7
Quality statement 6.2	5	4	4	13
**EXPERIENCE OF CARE TOTALS**	**32**	**21**	**20**	**73**
**STANDARD 7 (readiness)**	**24**	**12**	**10**	**46**
Quality statement 7.1	5	3	3	11
Quality statement 7.2	9	7	5	21
Quality statement 7.3	10	2	2	14
**STANDARD 8 (readiness)**	**32**	**4**	**6**	**42**
Quality statement 8.1	11	0	3	14
Quality statement 8.2	8	1	1	10
Quality statement 8.3	13	3	2	18

**STANDARD 2 (info systems)**	**8**	**6**	**3**	**17**
Quality statement 2.1	3	3	0	6
Quality statement 2.2	5	3	3	11
**READINESS TOTALS**	**64**	**22**	**19**	**105**
**GRAND TOTALS**	**162**	**109**	**85**	**356**

Step 3. Removal of duplicates and merging similar indicators

The fourth step involved removing duplicates sfor the provision of care and experience of care indicators. Indicators that were exact duplicates were deleted, but a record of all sources that included the same indicator with the exact same indicator definition was retained. There were many indicators that were conceptually similar, yet slightly different in terms of the scope of information captured. The study team convened through a series of meetings to compare all conceptually similar indicators, which had been grouped together under specific quality statements, and to select the indicator that *most comprehensively* captured the underlying construct of interest. After selecting the indicator for a given construct, all other similar indicators were hidden. We attempted to select one indicator where possible, but in some cases kept indicators that captured additional elements. Documentation of issues that should be considered during the scoring and final selection process were included in the notes for each selected indicator. At this stage, we also removed indicators that were clearly out of scope (e.g. indicators around homecare, bereavement, and positive memories around childbirth). After removing duplicates, we went from 623 to 168 provision of care indicators (Table S5), and 1497 to 264 experience of care indicators (Table S6).

Table S5. Number of Provision of Care indicators from the Scoping Review per Quality Statement

**Quality statement**	**Indicators after grouping by WHO Standards framework**	**Indicators after grouping and removing duplicates and consolidating similar indicators**
1.1a	33	15
1.1b	52	17
1.1c	107	n/a
1.2	10	4
1.3	14	5
1.4	54	23
1.5	8	4
1.6a	12	7
1.6b	103	21
1.7a	24	16
1.7b	8	3
1.8	35	n/a
1.9	38	22
**Total Standard 1 indicators**	**498**	**137**
3.1	14	6
3.2	14	8

3.3	9	5
Other standard 3	6	5
**Total Standard 3 indicators**	**43**	**24**
**Uncategorized**	**64**	**7**
**Total**	**605**	**168**

Note: there are 'n/a's for 1.1c and 1.8 values because these two quality statements were screened out

Table S6. Number of Experience of Care indicators per Quality Statement, based on scoping review organized by WHO quality standards

Quality statement	Indicators after grouping by WHO Standards framework	Indicators after grouping and removing duplicates
4.1	252	37
4.2	10	2
**Total Standard 4 Indicators**	**262**	**39**
5.1	185	38
5.2	312	30
5.3	214	50
**Total Standard 5 Indicators**	**711**	**138**
6.1	52	7
6.2	361	88
**Total Standard 6 Indicators**	**413**	**95**
**Uncategorized**	**141**	**12**
**Total**	**1527**	**264**

Section 4. Indicator Prioritization

Possible selection criteria were extracted from reports recommended by workstream members from other similar initiatives and exercises on selecting core indicators (e.g., Countdown to 2030, Nurturing Care Framework, Global Strategy for Women's, Children's and Adolescents' Health, ENAP, EPMM, etc). Documents identified in the scoping review were also checked for selection criteria ("criteria" was searched for in each document). Below is a list of resources used to identify selection criteria:

Boulkedid, R., Sibony, O., Goffinet, F., Fauconnier, A., Branger, B., & Alberti, C. (2013). Quality indicators for continuous monitoring to improve maternal and infant health in maternity departments: A modified Delphi survey of an international multidisciplinary panel. *PloS One*, 8(4), e60663. https://doi.org/10.1371/journal.pone.0060663

Countdown to 2030: Data Sources and Technical Review Process – 2016-2017.

Bunch, K.J., Allin, B., Jolly, M., Hardie, T., & Knight, M. (2018). Developing a set of consensus indicators to support maternity service quality improvement: using Core Outcome Set methodology including a Delphi process. *BJOG,* 125(12), 1612-1618

Diana, Mark L., Yeager, Valerie A., Hotchkiss, David R. (2017). Health Systems Strengthening: A Compendium of Indicators. USAID, Measure Evaluation. https://www.measureevaluation.org/resources/publications/tr-17-167b/at_download/document.

Chang, K.T., Hossain, P., Sarker, M., Montagu, D., Chakraborty, N.M., & Sprockett, A. (2020). Translating international guidelines for use in routine maternal and neonatal healthcare quality measurement. *Glob Health Action*, 13(1), 1783956.

Evans, S.M., Lowinger, J.S., Sprivulis, P.C., Copnell, B., & Cameron, P.A. (2008). Prioritizing quality indicator development across the healthcare system: identifying what to measure. *Internal Medicine Journal* 39: 648-654.

Global urban monitoring framework: A guide for urban monitoring of SDGs and NUA and other urban-related thematic or local, national and global frameworks.

Health Quality Ontario. (n.d.) How Indicators are Selected to Measure Ontario's Health System Performance.

Jolivet, R.R., et al. (2018). Ending preventable maternal mortality (EPMM): phase II of a multi-step process to develop a monitoring framework, 2016–2030. BMC Pregnancy and Childbirth 18:258. https://doi.org/10.1186/s12884-018-1763-8

Korst, L. M., Gregory, K. D., Lu, M. C., Reyes, C., Hobel, C. J., & Chavez, G. F. (2005). A Framework for the Development of maternal quality of care indicators. *Maternal and Child Health Journal*, 9(3), 317–341. https://doi.org/10.1007/s10995-005-0001-y

Kotter, T., Blozik, E., & Scherer, M. (2012). Methods for the guideline-based development of quality indicators – a systematic review. Implementation Science 2012, 7:21 http://www.implementationscience.com/content/7/1/21

Lattof, S.R., Moran, A.C., Kidula, N., Moller, A.B., Jayathilaka, C.A., Diaz, T., & Tunçalp, Ö. (2020). Implementation of the new WHO antenatal care model for a positive pregnancy experience: a monitoring framework. BMJ Glob Health, 5(6).

Lazzaretto, E., Nespoli, A., Fumagalli, S., Colciago, E., Perego, S., Locatelli, A. (2018). Intrapartum care quality indicators: a literature review. *Minerva Ginecol*, 70(3), 346-356.

Marshall, M., Leatherman, S., & Mattke, S. (2004). Selecting Indicators for the Quality of Health Promotion, Prevention and Primary Care at the Health Systems Level in OECD Countries. OECD Health Technical Papers No. 16. https://dx.doi.org/10.1787/627626833816

Newborn Measurement Review Template for Kangaroo Mother Care Working Group

NHS. The Good Indicators Guide: Understanding how to use and choose indicators.

Prioritization of paediatric quality of care indicators: A concept note.

Redshaw, M., Martin, C.R., Savage-McGlynn, E., Harrison, S. (2019). Women's experiences of maternity care in England: preliminary development of a standard measure. BMC Pregnancy Childbirth, 19(1), 167

Rich, R., D'Hont, T., Linton, J., Murphy, K.E., Veillard, J., & Chatwood, S. (2016). Performance indicators for maternity care in a circumpolar context: a scoping review. *Int J Circumpolar Health,* 75, 31470.

Saturno-Hernández, P.J., Martínez-Nicolás, I., Moreno-Zegbe, E., Fernández-Elorriaga, M., Poblano-Verástegui, O. (2019). Indicators for monitoring maternal and neonatal quality care: a systematic review. *BMC Pregnancy Childbirth*, 19(1), 25.

World Health Organization. (2014). Newborn health: draft action plan. *Sixty-seventh World Health Assembly Provisional agenda item 14.2.*

World Health Organization. (2018). Standards for improving the quality of care for children and young adolescents in health facilities.

World Health Organization. (2015). Global reference list of 100 core health indicators.

World Health Organization. (2019). Quality of Care for Maternal and Newborn Health: A Monitoring Framework for Network Countries. WHO.

Women and Health Initiative. (2020). Improving maternal health measurement capacity and use (IMHM): Additional Indicator Development Technical Consultations | May 11-July 15, 2020

Wool C; Kain VJ; Mendes J; Carter BS. (2018). Quality predictors of parental satisfaction after birth of infants with life-limiting conditions. *Acta Paediatr*, 107(2), 276-282.

Agency for Healthcare Research and Quality tool.

Every Newborn Action Plan.

For each proposed selection criterion, the study team extracted definitions and information on how it was used for other initiatives. Criteria with similar definitions were grouped together in an excel spreadsheet. The study team reviewed the criteria and developed a proposal on which criterion to use for the EmONC indicators. The study team presented the full criteria list and the shorter proposed list to the quality of care workstream for discussion. Consensus was reached on four selection criteria (relevant/important, actionable, feasible, evidence-based) (Table S7). The group also agreed to exclude any indicators that had been **invalidated.**

Table S7. Selection criteria for scoring the provision and experience of care indicators

Selection criteria	Definition
Relevant/important	Domain areas is reflected in global guidance, relates to leading causes of maternal and newborn deaths, and is relevant to emergency obstetric care*<br><br>Leading causes of maternal death (Say et al., 2014):<br>Obstetric haemorrhage<br>Pregnancy-related sepsis<br>Hypertensive disorders (pre-eclampsia and eclampsia)<br>Abortion (induced abortion, miscarriage, ectopic pregnancy)<br>Embolism<br>Other direct causes (thromboembolism, uterine inversion or rupture, suicide, obstructed labour)<br>Indirect causes (infectious disease, cardiac disease, liver disease)<br><br>Leading causes of neonatal death (Vos et al., 2015; Perin et al., 2021):<br>Pre-term birth complications<br>Intrapartum-related events (includes birth asphyxia and birth trauma)<br>Congenital abnormalities (ex. Neonatal encephalopathy)<br>Lower respiratory infections (pneumonia)<br>Diarrhoea<br>Tetanus<br>Sepsis or meningitis<br>Other conditions
Actionable	Data for the indicator is assessed at a frequent interval/provides data that can help inform a programmatic or policy level response.
Feasible	Indicator is already being collected in SPA, SARA or in other routine data collection or is feasible for collection in LMICs
Evidence-based	Indicator captures information on an intervention or practice proven effective at improving health/reducing mortality

Note: *relevance to emergency obstetric care only considered for provision of care indicators

Options for criteria application and generating core indicators:

The original plan for applying the criteria and selecting the core indicators was as follows:

- Members of the study team would individually score each provision of care and experience of care indicator using the four selection criteria: relevant/important, actionable/useful, feasible, evidence-based. For each selection criterion, the individual study team member was tasked with assigning a score between 0-2 (where 0 corresponds to low, 1 medium and 2 high). Each indicator could, therefore, receive a total score value ranging from a minimum of 0 to a maximum of 8.

- After completing the scoring of all indicators grouped under a given quality statement, individual team members would then review these scores and select the top indicator for each quality statement.
- Team members would provide comments for each indicator describing the rationale used for determining the score.
- The scores submitted from each team member would then be compiled into one excel file to allow for comparison and review. The aim of the review and comparison step was to check for inter-rater reliability, to discuss indicators for which there were wide variations in scores across reviewers, establish a threshold level for removing indicators, and to apply this threshold value to arrive at a reduced set of indicators (one for each of the 21 quality statements – or 14 provision of care and 7 experience of care indicators). This reduced set of indicators was then to be shared and discussed with the quality of care working group, resulting in consensus on a recommended core set for the EmONC handbook.

The first steps were executed according to plan, starting with the provision of care indicators. However, when the study team convened to review the compiled individual score submissions for these indicators, wide variations in the scoring made evident that there were flaws with the approach. Scoring differences between study team members were a result of the way the indicators had been grouped by specific quality statements and then further merged to generate a comprehensive indicator for capturing the underlying construct of the quality statement (Step 3). For example, under Quality statement 1.1a: Women are assessed routinely on admission and during labour and childbirth and are given timely, appropriate care, some indicators were specific to practices provided during one phase of the labor and childbirth cycle (e.g., upon arrival or immediately after birth) while others were composite covering all phases. Some reviewers scored the more specific indicators lower than the composite measures because of lack of completeness, while others scored all indicators the same.

Consistency in the documentation provided by study members, however, resulted in agreement for an alternative approach to indicator prioritization by generating composite measures for each of the quality standards potentially with an associated checklist. Study members returned to the quality statements retained for both provision of care (14 out of the original 16) and experience of care (7 out of the original 7) and to re-group them into "maternal quality statements" and "newborn quality statements" (to ensure adequate representation of newborn indicators). The results were as follows:

- Provision of care (Table S8) re-organized:
    - 10 maternal quality statements
    - 8 newborn quality statements
- Experience of care (Table S9) re-organized:
    - 7 maternal quality statements
    - 2 newborn quality statements

Table S8. Provision of care quality statements re-organized

Maternal Quality Statements	Newborn Quality Statements
Quality statement 1.1a: Women are assessed routinely on admission and during labour and childbirth and are given timely, appropriate care.	Quality statement 1.1b: Newborns receive routine care immediately after birth.
Quality statement 1.2: Women with pre-eclampsia or eclampsia promptly receive appropriate interventions.	
Quality statement 1.4: Women whose progress in labour is delayed or whose labour is obstructed receive appropriate interventions, according to WHO guidelines.	
	Quality statement 1.5: Newborns who are not breathing spontaneously receive appropriate stimulation and resuscitation with a bag-and-mask within 1 min of birth, according to WHO guidelines.
Quality statement 1.6a: Women in preterm labour receive appropriate interventions for both themselves and their babies, according to WHO guidelines.	Quality statement 1.6b: Preterm and small babies receive appropriate care, according to WHO guidelines. [ES]
Quality statement 1.7a: Women with or at risk for infections during labour, childbirth or the early postnatal period promptly receive appropriate interventions, according to WHO guidelines.	Quality statement 1.7b: Newborns with suspected infection or risk factors for infection are promptly given antibiotic treatment, according to WHO guidelines.
	Quality statement 1.9b: No newborn is subjected to unnecessary or harmful practices during childbirth and the early postnatal period.
Quality statement 3.1a: Every woman is appropriately assessed on admission, during labour and in the early postnatal period to determine whether referral is required, and the decision to refer is made without delay.	Quality statement 3.1b: Every newborn is appropriately assessed to determine whether referral is required, and the decision to refer is made without delay.
Quality statement 3.2a: For every woman who requires referral, the referral follows a pre-established plan that can be implemented without delay at any time.	Quality statement 3.2b: For every newborn who requires referral, the referral follows a pre-established plan that can be implemented without delay at any time.
Quality statement 3.3a: For every woman referred within or between health facilities, there is appropriate information exchange and feedback to relevant health care staff.	Quality statement 3.3b: For every newborn referred within or between health facilities, there is appropriate information exchange and feedback to relevant health care staff.

Table S9. Experience of care Quality Statements re-organized

Domain	Maternal Quality Statement	Newborn Quality Statement
Communication	Quality statement 4.1: All women and their families receive information about the care and have effective interactions with staff.	
	Quality statement 4.2: All women and their families experience coordinated care, with clear, accurate information exchange between relevant health and social care professionals.	
Autonomy	Quality statement 5.3: All women can make informed choices about the services they receive, and the reasons for interventions or outcomes are clearly explained.	
Privacy and confidentiality	Quality Statement 5.1a: All women have privacy around the time of labour and childbirth, and their confidentiality is respected.	Quality Statement 5.1b: All newborns have their confidentiality respected.
Dignified care	Quality statement 5.2a: No woman is subjected to mistreatment, such as physical, sexual or verbal abuse, discrimination, neglect, detainment, extortion or denial of services.	Quality statement 5.2b: No newborn is subjected to mistreatment, such as physical, sexual or verbal abuse, discrimination, neglect, detainment, or denial of services.
Companion of choice	Quality statement 6.1: Every woman is offered the option to experience labour and childbirth with the companion of her choice.	
Supportive care	Quality statement 6.2: Every woman receives support to strengthen her capability during childbirth.	

The team then developed three potential scenarios for creating a core set of provision of care indicators for the EmONC handbook to be discussed with the broader quality of care working group:

- <u>Checklist approach</u>: Create a composite measure for each of the quality statements to best capture the construct. Assessment would be a scoring modality (x out of y)
  - Comprehensive check list
  - Parsimonious check list
- <u>Proxy approach</u>: Selection of proxies for the construct selected based on feasibility of measurement plus relevance/importance
  - One proxy

◦ More than one proxy (depending upon complexity of underlying construct)

- Combined approach:

◦ Checklist for routine care, proxies for specific complications

Three examples based on key quality statements were generated for provision of care indicators.

1) Indicator capturing routine care across phases of care from admission to immediately after delivery/discharge

Quality statement 1.1a: Women are assessed routinely on admission and during labour and childbirth and are given timely, appropriate care.

2) Indicator related to a specific leading cause of maternal death

Quality statement 1.4: Women whose progress in labour is delayed or whose labour is obstructed receive appropriate interventions, according to WHO guidelines.

3) Indicator related to care for small and sick newborns

Quality statement 1.6b: Preterm and small babies receive appropriate care, according to WHO guidelines.

The three options were then presented to the full quality of care workstream for decision. Consensus was reached to use the proxy approach for provision of care, with the exception of using the composite approach for the routine care indicator.

It was agreed that the experience of care indicators would need to be derived from the Person-Centered Maternity (PCMC) scale (Afulani et al., 2017).

Step 6. Identifying proxy indicators for provision of care.

For each quality statement, the study team went through the categorized indicators and selected one to three proxies using the criteria of relevant/important and feasible (Table S10). We opted to select indicators capturing information on tasks that should be performed and did not select indicators covering harmful practices (quality statement 1.9), which could be included in the effort underway on defining a set of routine signal functions. We selected one indicator (prevention of mother-to-child transmission of HIV) that was not categorized by a WHO standard or quality statement. In the guidance document, there may need to be a section that provides guidance for special populations, which may be more prevalent/relevant for certain regions or countries.

Step 7. Sharing of proxy indicators for provision of care with the quality of care workstream.

We shared the selected indicators via email with the full quality of care workstream for written feedback. We reviewed all feedback received for each indicator, and modified the indicators as needed (modifications and comments documented in the Notes column of Table S10).

Where possible, we aligned our indicator definitions with the indicators in the Quality, Equity, Dignity (QED) indicator catalogue (QED Network, 2019). In cases where our selected indicators were more

comprehensive than the QED catalogue, we used the more comprehensive indicator while still making sure the QED wording/terminology was retained as possible. AMDD has commissioned research on referral systems, which is being led by Loveday Penn-Kekana. We will align the quality of care indicators on referrals with her findings once they are ready. We suggest that stratification by mode of delivery be a consideration for all indicators as appropriate/relevant.

Table S10. Prioritized provision of care of indicators

Quality statement	Indicator Definition and Number	Numerator and Denominator	Notes
**Standard 1: Every woman and newborn receives routine, evidence-based care and management of complications during labour, childbirth and the early postnatal period, according to WHO guidelines**			
1.1a: Women are assessed routinely on admission and during labour and childbirth and are given timely, appropriate care.	1. % women who gave birth in the health facility whose blood pressure, pulse, temperature, vaginal examination, and fetal heart sounds were taken during labour, childbirth, and the early postpartum period.***	Numerator: Number of women who gave birth in the health facility whose blood pressure, pulse, temperature, vaginal examination, and fetal heart sounds were taken during labour, childbirth, and the early postpartum period (indicator can be disaggregated by each of these stages).<br><br>Denominator: Number of women who gave birth in the health facility.	Vaginal examination and fetal heart sound measurement were added as additional assessments to the WHO standard.<br><br>Ideally this indicator is observed; otherwise taken from records.<br><br>Recognizing that observation is not always possible, we have changed the wording from “appropriately recorded” to “taken”.<br><br>Ideally, we would have one indicator per stage (e.g., admission, during labour, during childbirth), scored from 0-5.
1.1b: Newborns receive routine care immediately after birth.	2. % newborns who received all four elements of essential newborn care: immediate and thorough drying, immediate skin-to-skin contact, delayed cord clamping and initiation of breastfeeding in the first hour.***	Numerator: Number of newborns who received all four elements of essential newborn care.<br><br>Denominator: Number of newborns.	Scored from 0-4.
1.2: Women with pre-eclampsia or	3.% women with severe pre-eclampsia or eclampsia in the	Numerator: Number of women with severe pre-	To address the fact that a full dose can mean a loading dose with or

eclampsia promptly receive appropriate interventions.	health facility who received magnesium sulfate.***	eclampsia or eclampsia who delivered in the health facility who were treated with magnesium sulfate.<br><br>Denominator: Number of women who delivered in the health facility with severe pre-eclampsia or eclampsia.	without a maintenance dose, we propose two nuanced indicators for consideration:<br><br>% women with severe pre-eclampsia or eclampsia in the health facility who received the first loading dose; and maintenance dose of magnesium sulfate if needed.<br><br>% women with severe pre-eclampsia or eclampsia in the health facility who required a second dose who received it.
	4. % women with severe hypertension in pregnancy in the health facility who received the recommended antihypertensives.	Numerator: Number of women with severe hypertension in pregnancy admitted to the health facility who received recommended antihypertensives.<br><br>Denominator: Number of women with severe hypertension in pregnancy who were admitted to the health facility.	We are recommending a measure related to magnesium sulfate. Do we want more than one indicator related to pre-eclampsia/eclampsia? Note that there are some contraindications to antihypertensives.<br><br>A working group member suggested removal of this indicator, but further discussion is needed on whether to remove this indicator since it is recommended as part of standard of care. Further discussion confirmed keeping this indicator.
1.3: Women with post-partum haemorrhage promptly receive	5.% women with post-partum haemorrhage in the health facility who received	Numerator: Number of women with post-partum haemorrhage who delivered in the health facility who	Change the target population to all women with PPH and not just those with PPH due to atony.

appropriate interventions.	therapeutic uterotonic drugs.***	received therapeutic uterotonic drugs.<br><br>Denominator: Number of women with post-partum haemorrhage who delivered in the health facility.	Define timeframe – 1 minute? This requires a first assessment of the cause of PPH. There are nuances for administration of therapeutic uterotonics and for PPH that are different from prophylactic administration of a uterotonic as part of active management of third stage of labor.
	6.% women in the health facility with post-partum haemorrhage due to a retained placenta for whom manual removal of the placenta was performed.	Numerator: Number of women who delivered in the health facility with post-partum haemorrhage due to a retained placenta for whom manual removal of the placenta was performed.<br><br>Denominator: Number of women who delivered in the health facility with post-partum haemorrhage due to a retained placenta.	This requires first assessment of the cause of PPH.<br><br>We have dropped the skilled birth attendant specification as it is unlikely that untrained staff will perform manual removal.<br><br>Note: We have two indicators related to PPH, but due to different causes. If the group agrees, we could limit to one indicator, either this one or the uterotonic. However, this indicator on manual removal is related to a signal function that will be part of the Delphi.
1.4: Women whose progress in labour is delayed or whose labour is obstructed receive appropriate interventions,	7.% women in the health facility with prolonged and/or obstructed labour who gave birth by caesarean section.***	Numerator: Number of women with prolonged/obstructed labour who delivered in the health facility who gave birth by caesarean section.	Suggest limit to just obstructed labour. If limiting to just this, the goal would be 100% coverage. There are other options for prolonged labor than c-section (i.e., assisted delivery). For obstructed labor, c-section is

according to WHO guidelines.		Denominator: Number of women with prolonged/obstructed labour who delivered in the health facility.	indicated. We have a second indicator below for prolonged labor.
	8.% women with delayed second stage of labour who underwent instrumental vaginal birth or c-section (disaggregate by mode of delivery).	Numerator: Number of women with delayed second stage of labour who gave birth in the health facility who underwent instrumental vaginal birth or c-section (disaggregate by mode of delivery).<br><br>Denominator: Number of women with delayed second stage of labour who delivered in the health facility.	Modified WHO Standards wording.
	9. % women in the health facility with confirmed delay in progress of the first stage of labour who received oxytocin for augmentation.	Numerator: Number of women who delivered in the health facility with confirmed delay in progress of the first stage of labour who received oxytocin for augmentation.<br><br>Denominator: Number of women who delivered in the health facility with confirmed delay in progress of the first stage of labour.	Different denominators – women with epidural, high low risk.<br><br>Discussion needed on issues around measuring confirmed delay (in context of partograph discussions) and women's ability to choose augmentation or not.
1.5: Newborns who are not breathing	10.% newborns who were not breathing spontaneously	Numerator: Number of newborns (live births and	Updated to align with ENAP indicator.

spontaneously receive appropriate stimulation and resuscitation with a bag-and-mask within 1 min of birth, according to WHO guidelines.	who received any positive pressure ventilation using any device (most commonly bag and mask).***	stillbirths excluding macerated stillbirths) who were not breathing spontaneously who received any positive pressure ventilation using any device (most commonly with bag and mask)<br><br>Denominator: Number of newborns (live births and stillbirths excluding macerated stillbirths) who were not breathing spontaneously.	
1.6a: Women in preterm labour receive appropriate interventions for both themselves and their babies, according to WHO guidelines.	11.% women with preterm pre-labour rupture of membranes who gave birth in the health facility who received prophylactic antibiotics.	Numerator: Number of women with preterm pre-labour rupture of membranes who gave birth in the health facility who received prophylactic antibiotics.<br><br>Denominator: Number of women with preterm pre-labour rupture of membranes who gave birth in the health facility.	
	12.% women who gave birth in the facility between 24 and 34 weeks gestational age who received at least one dose of antenatal corticosteroids (ACS).***	Numerator: Number of women who gave birth in the facility between 24 and 34 weeks gestational age and received at least one dose of ACS.	Updated with ENAP indicator.

		Denominator: Number of women who gave birth in the facility between 24 and 34 weeks gestational age.	
1.6b: Preterm and small babies receive appropriate care, according to WHO guidelines.	13.% live-born low-birth-weight (<2500g) newborns born in the health facility who are initiated on kangaroo mother care (KMC)** or admitted to a KMC unit if a separate unit exists.***	Numerator: Number of admitted low birth weight newborns (< 2500g) who are initiated on KMC anywhere in the facility (disaggregate by <2000 g where possible).<br><br>Denominator: Number of admitted low birth weight newborns (<2500g) (disaggregate by <2000g where possible).	Initiation vs continuous. Also think about indicator for breastfeeding support.<br><br>Updated to align with ENAP recommendations.
1.7a: Women with or at risk for infections during labour, childbirth or the early postnatal period promptly receive appropriate interventions, according to WHO guidelines.	11.% women who gave birth in the health facility with preterm pre-labour rupture of membranes who received prophylactic antibiotics.+	Numerator: Number of women who gave birth in the health facility with preterm pre-labour rupture of membranes who received prophylactic antibiotics.<br><br>Denominator: Number of women who gave birth in the health facility with preterm pre-labour rupture of membranes.	Same as indicator under 1.6a.
	14.% women who gave birth in the health facility with signs of infection treated	Numerator: Number of women who gave birth in the health facility with signs	Removed “injectable” specification

	with appropriate antibiotics.***	of infection who received appropriate antibiotics.<br><br>Denominator: Number of women who gave birth in the health facility with signs of infection.	
1.7b: Newborns with suspected infection or risk factors for infection are promptly given antibiotic treatment, according to WHO guidelines.	15.% newborns identified as cases of possible serious bacterial infection in outpatient settings or clinically suspected sepsis in inpatient settings who received at least two days of appropriate injectable antibiotics.***	Numerator: Number of newborns identified as cases of possible serious bacterial infection in outpatient settings or clinically suspected sepsis in inpatient settings who received at least two days of appropriate injectable antibiotics.<br><br>Denominator: Number of newborns identified as cases of possible serious bacterial infection in outpatient settings or clinically suspected sepsis in inpatient settings.	Make more specific. Denominators – signs of infection vs symptomatic for syphilis.<br><br>Updated to align with ENAP recommendations.
**Standard 3: Every woman and newborn with condition(s) that cannot be dealt with effectively with the available resources is appropriately referred.**			
3.1: Every woman and newborn is appropriately assessed on admission, during labour and in the early postnatal	16. % sick, preterm or small newborns who could not be managed at the health facility who were transferred to an appropriate level of care within 1 hour of a decision, accompanied by a	Numerator: Number of sick, preterm or small newborns who could not be managed at the health facility who were transferred to an appropriate level of care within 1 hour of a decision,	We will align with recommendations coming out of referral consultancies.

period to determine whether referral is required, and the decision to refer is made without delay.	health care professional and a completed standardized referral note.***	accompanied by a health care professional and a completed standardized referral note.<br><br>Denominator: Number of sick, preterm or small newborns who could not be managed at the health facility.	
	17. % pregnant or postnatal women who could not be managed at the health facility who were transferred to a higher-level facility for childbirth or further management without delay, accompanied by a health care professional and a completed standardized referral note.***	Numerator: Number of pregnant or postnatal women who could not be managed at the health facility who were transferred to a higher-level facility for childbirth or further management without delay, accompanied by a health care professional and a completed standardized referral note.<br><br>Denominator: Number of pregnant or postnatal women who could not be managed at the health facility.	We will align with recommendations coming out of referral consultancies.
3.2: For every woman and newborn who requires referral, the referral	18. % pregnant and postnatal women and newborns who were referred with appropriate emergency	Numerator: Number of pregnant and postnatal women and newborns who were referred with	Changed wording from negative to positive ("without emergency transport" to "with emergency transport"). Recommend

follows a pre-established plan that can be implemented without delay at any time.	transport (disaggregate by pregnant woman, postnatal woman, newborn)	appropriate emergency transport (disaggregate by pregnant woman, postnatal woman, newborn).<br><br>Denominator: Number of pregnant and postnatal women and newborns who were referred (disaggregate by pregnant woman, postnatal woman, newborn).	disaggregation by maternal + newborn. Related to readiness. Can be stratified into those who develop complications/died and those who did not.<br><br>We will align with recommendations coming out of referral consultancies.
3.3: For every woman and newborn referred within or between health facilities, there is appropriate information exchange and feedback to relevant health care staff.	19. % referred women and newborns seen at the referring facility who received timely care at the referral facility (disaggregate by women, newborns).	Numerator: Number of referred women and newborns seen at the referring facility who received timely care at the referral facility (disaggregate by women, newborns).<br><br>Denominator: Number of referred women and newborns seen at the referring facility (disaggregate by women, newborns).	Define timely care (depends on emergency). Ex. Within 15 mins?<br><br>We will align with recommendations coming out of referral consultancies.
**Uncategorized: Special populations**			
Not listed in the WHO Standards	20. % women living with HIV who delivered in the health facility and received appropriate prophylaxis to prevent mother-to-child	Numerator: Number of women living with HIV who delivered in the health facility who received appropriate prophylaxis to prevent mother-to-child	Propose having a special populations section. For now, just have HIV, but may have different special populations for different regions (ex. malaria).

	transmission and antiretroviral therapy.	transmission and antiretroviral therapy.<br><br>Denominator: Number of women living with HIV who delivered in the health facility.	

Note: We considered core references as the WHO Standards, QED catalogue and ENAP metric recommendations.

*All indicators should be measured based on a specified time period.

**Kangaroo mother care is defined as care of preterm infants carried skin-to-skin with the mother. Key features include continuous skin-to-skin contact with the mother, exclusive breastfeeding, and early discharge from hospital in the kangaroo position with frequent home visits by health workers [34].

*** Indicators identified as collectable through routine information systems.

+ duplicate indicator that was categorized under two WHO Standards.++ Note: A clinical trial recently published in the New England Journal of Medicine has provided evidence for the effectiveness of a bundle of treatments for postpartum hemorrhage (https://www.nejm.org/doi/full/10.1056/NEJMoa2303966). For clinical management purposes, the following composite indicator is important to regularly monitor: % women with post-partum haemorrhage in the health facility who received a bundle of first-response treatments (uterine massage, oxytocic drugs, tranexamic acid, intravenous fluids, examination, and escalation). The proxy provision of care measure selected for postpartum hemorrhage: % of women with post-partum haemorrhage in the health facility who received therapeutic uterotonic drugs is still appropriate as a red-flag indicator for district level monitoring. If coverage is low, this is an indication that the quality of care for PPH needs to be addressed including the full bundle of treatments. If the coverage of uterotonics is high, but PPH related mortality remains high, this will trigger investigation into the full bundle of treatments. As measurement of the bundle improves, aggregated data could eventually be reported to the district level.

Step 7. Identifying items for experience of care scale

We took a similar approach to prioritizing the experience of care indicators in that we grounded our process on an existing reference document. However, instead of the WHO Standards document, we used the Person-Centered Maternity (PCMC) scale (Afulani et al., 2017), which has been validated for measuring experience of care in various LMICs (Afulani et al., 2018; Afulani et al., 2019; Özşahin et al., 2021; Rishard et al., 2021) and the PCMC-US scale (Afulani et al., 2022). Of note, these "indicators" were mostly survey items pulled from various questionnaires. We therefore went through the categorized indicators for experience of care and noted whether the indicator corresponded to an original PCMC item or to an additional item of the US version of the PCMC scale. We also compared these indicators to the corresponding PCMC item to check whether the PCMC items required updating. We identified indicators that were not captured by the PCMC scale and selected those that were as important/relevant (note that we did emphasize relevance to emergency obstetric care as much) and feasible as additional items that could be measured to fully capture women's and newborns' experience of care.

Step 8. Sharing of experience of care scale and additional items with workstream

We shared the experience of care scale and additional items with the workstream for feedback. We added 3 additional items in response to feedback.

Recognising the need for a parsimonious set of indicators in the EmONC handbook, we recommend the PCMC score, which ranges from 0 to 100, where higher scores represent more positive experiences. This score will ideally be measured with the 30-item PCMC scale. Where it is not practical to use the 30-item scale, we recommend the shorter 13-item version that has also been psychometrically validated (Afulani et al., 2019). However, this 13-item version excludes the items on birth companionship and verbal and physical abuse, which we recommend including as additional items because they are important aspects of women's experiences. The PCMC scale has been included in the newly revised SPA questionnaire, and so use of this scale for EmONC assessments will ensure alignment with data collected through the SPA tools. Where in-depth efforts on women's experiences is a goal, we have provided additional items that could be used in addition to the PCMC scale as needed.

Table S11. Prioritized experience of care indicators

**PCMC 30-item scale**	**Included in the 13-item scale? Yes or No**
**Supportive care**	
1. How did you feel about the amount of time you waited?	No
2. Were you allowed to have someone you wanted to stay with you during labor?	No
3. Were you allowed to have someone you wanted to stay with you during the delivery?	No
4. When you needed help, did you feel the doctors, nurses, midwives, or other staff at the facility paid attention?	Yes
5. Did the doctors, nurses, midwives, or other staff at the facility talk to you about how you were feeling?	Yes

6. Did the doctors, nurses, midwives, or other staff at the facility support your anxieties and fears?	No
7. Do you feel the doctors, nurses, midwives or other staff did everything they could to help control your pain?	No
8. Did you feel the doctors, nurses, midwives, or other staff at the facility took the best care of you?	Yes
9. Did you feel you could completely trust the doctors, nurses, midwives, or other staff at the facility with regards to your care?	No
10. Thinking about the wards, washrooms, and the general environment of the health facility, will you say the facility was very clean, clean, dirty, or very dirty?	No
11. Do you think there was enough health staff in the facility to care for you?	No
12. Thinking about the labor and postnatal wards, did you feel the health facility was crowded? (revised wording: Did you feel the place you gave birth was crowded during your birth stay? (e.g., not enough beds, moved from room to room, being in triage a long time)	No
13. In general, did you feel safe in the health facility?	No
14. Was there water in the facility?	No
15. Was there electricity in the facility?	No
**Dignity and respect**	
16. Did the doctors, nurses, midwives, or other staff at the facility treat you with respect	Yes
17. Did the doctors, nurses, midwives, and other staff at the facility treat you in a friendly manner?	Yes
18. During examinations in the labor room, were you covered up with a cloth or blanket or screened with a curtain so that you did not feel exposed?	Yes
19. Do you feel like your health information was or will be kept confidential at this facility?	No
20. Did you feel the doctors, nurses, midwives, or other health providers shouted at you, scolded, insulted, threatened, or talked to you rudely?	No
21. Did you feel like you were treated roughly… like pushed, beaten, slapped, pinched, physically restrained, or gagged?	No
**Communication and autonomy**	
22. During your time in the health facility did the doctors, nurses, midwives, or other health care providers introduce themselves to you when they first came to see you?	No
23. Did the doctors, nurses, midwives, or other health care providers call you by your preferred name?	Yes
24. Did you feel like the doctors, nurses, midwives, or other staff at the facility involved you in decisions about your care?	Yes
25. Did the doctors, nurses, midwives or other staff explain to you why they were doing examinations or procedures on you?	Yes
26. Did the doctors, nurses, midwives or other staff explain to you why they were giving you any medicine?	Yes
27. Did the doctors, nurses, midwives, or other staff at the facility ask your permission/consent before doing procedures and examinations on you?	Yes

28. During the delivery, do you feel like you were able to be in the position of your choice?	Yes
29. Did the doctors, nurses, midwives, or other staff at the facility speak to you in a language or in terms you could understand.	No
30. Did you feel you could ask the doctors, nurses, midwives, or other staff at the facility any questions you had	Yes

Notes: Notes: Items from the 30-item PCMC scale are measured on a 4-point scale from 0 to 3 (E.g, 0, No never; 1, Yes, a few times; Yes, most of the time; Yes, all the time).

Table S12. Additional 29 experience of care items, organized by mother and newborn

**Additional experience of care items**	**Source**
Mother	
1. The proportion of all women discharged from the labour and childbirth area of the facility who reported receiving written and verbal information and counselling on the following elements before discharge: nutrition and hygiene, birth spacing and family planning, exclusive breastfeeding and maintaining lactation, keeping their baby warm and clean, communication and play with the baby, danger signs for the mother and newborn and where to go in case of complications.	WHO Standards [4]
2. The proportion of all women who gave birth in the health facility who reported that they were given the opportunity to discuss their concerns and preferences.	WHO Standards [4]
3. The proportion of women who reported that they were told different things by different care providers about their health that led to confusion.	Wong 2013 [35]
4. The proportion of all women who gave birth in the health facility who reported that health care staff showed good knowledge of their history and the care that had been given to date.	WHO Standards [4]
5. Proportion of women who reported that health providers sexually harassed them or made sexual advances (for example, inappropriate touching or sexual comments that make them feel uncomfortable).	Freedman 2018 [36]
6. The proportion of mothers who reported not being cleaned after birth and third stage of labour.	Banks 2018 [37]
7. The proportion of women who reported being instructed to clean up blood, urine, faeces or amniotic fluid.	Bohren 2018 [38]
8. The proportion of women who reported being denied care for any reason.	Afulani 2020 [9]
9. The proportion of women who reported being detained at facilities due to lack of payment.	Afulani 2020 [9]

10. The proportion of women who gave birth in the health facility who reported being aware of the existence and location of a complaints box.	WHO Standards [4]
11. The proportion of all women in the health facility who made a complaint whose complaints were acted upon without repercussions.	WHO Standards [4]
12. The proportion of procedures in the health facility that require written consent for which there is an associated record of consent signed by the woman or a family member.	WHO Standards [4]
13. The proportion of carers in the health facility who report having received information about the care plan for their newborn.	Recommendation submitted for the SPA revision process [10]
14. The proportion of parents who reported feeling that the staff at the local referring hospital explained the reason for transfer of their baby (only applies if baby was transferred from another unit).	Thyagarajan 2018 [39]
15. The proportion of mothers (carers) who reported being supported in family-centered care (facility allows companion/ family member, there is place for family member to sleep, place for family member to eat, place for family member to bathe).	Recommendation submitted for the SPA revision process [10]
16. The proportion of women who reported having direct access to the bathroom in the room they were lying after giving birth.	Baranowska 2020 [39]
17. The proportion of women with disabilities who reported that the hospital, clinic, or healthcare provider(s) office was accessible given their needs (e.g. specialized equipment, extra space).	Wong 2013 [35]
18. The proportion of all women undergoing bereavement or an adverse outcome who reported receiving additional emotional support from health facility staff.	WHO Standards [4]
19. The proportion of women who reported being discharged too early after birth.	Ziabakhsh 2018 [40]
20. The proportion of women who reported being separated from their baby without medical indication.	Azhar 2018 [41]
21. The proportion of women who reported being encouraged and/or able to mobilize during labor.	Bohren 2018 [38]
22. The proportion of all healthy mothers on postnatal wards or areas in the health facility who reported receiving breastfeeding counselling and support from a skilled health care provider.	WHO Standards [4]

23. The proportion of women who reported being asked for bribes or payments other than the official payment.	Afulani 2018 [24]
24. The proportion of women who reported that they were treated differently because of any personal attribute such as their age, marital status, number of children, education, wealth, sexual orientation, race/ethnicity/tribe, connections with the facility, etc..	Afulani 2022 [23]
Newborn	
25. The proportion of women who reported feeling that their newborn's health information was or would be kept confidential at the facility.	Adapted from WHO Standards [4]
26. Proportion of women who reported their newborns were maltreated.	Recommendation submitted for the SPA revision process [10]
27. The proportion of newborns who had prompt removal of soiled wrapper or diaper and cleaning of urine and faeces.	Sacks 2017 [42]
28. The proportion of carers in the health facility who report having received information about the care plan for their newborn.	Recommendation submitted for the SPA revision process [10]
29. The proportion of carers of small and sick newborns who reported receiving appropriate developmental supportive care for the newborn during their stay in the health facility.	Recommendation submitted for the SPA revision process [10]

Table S13. Sources of prioritized indicators

Title	Author	Date	Location	Type
Disrespect and abuse during childbirth in district Gujrat, Pakistan: A quest for respectful maternity care. PloS One.	Azhar Z, Oyebode O, Masud H.	2018	Pakistan	Peer-reviewed literature
A Rapid Review of Available Evidence to Inform Indicators for Routine Monitoring and Evaluation of Respectful Maternity Care.	Afulani PA, Buback L, McNally B, Mbuyita S, Mwanyika-Sando M, Peca E.	2020	Multi-country	Peer-reviewed literature
Jeopardizing quality at the frontline of healthcare: prevalence and risk factors for disrespect and abuse during facility-based childbirth in Ethiopia.	Banks KP, Karim AM, Ratcliffe HL, Betemariam W, Langer A.	2018	Ethiopia	Peer-reviewed literature

What are the Critical Elements of Satisfaction and Experience in Labor and Childbirth-A Cross-Sectional Study.	Baranowska B, Kajdy A, Pawlicka P, Pokropek E, Rabijewski M, Sys D, et al.	2020	Poland	Peer-reviewed literature
Methodological development of tools to measure how women are treated during facility-based childbirth in four countries: labor observation and community survey.	Bohren MA, Vogel JP, Fawole B, Maya ET, Maung TM, Baldé MD, et al.	2018	Multi-country (Ghana, Guinea, Myanmar, Nigeria)	Peer-reviewed literature
Observation versus self-report in the measurement of disrespect and abuse during facility-based childbirth.	Freedman LP, Kujawski SA, Mbuyita S, Kuwawenaruwa A, Kruk ME, Ramsey K, et al.	2013	Tanzania	Peer-reviewed literature
Defining disrespect and abuse of newborns: a review of the evidence and an expanded typology of respectful maternity care.	Sacks, E.	2017	Multi-country	Peer-reviewed literature
Parental perceptions of hypothermia treatment for neonatal hypoxicischaemic encephalopathy.	Thyagarajan, B.	2018	United Kingdom	Peer-reviewed literature
Voices of Postpartum Women: Exploring Canadian Women's Experiences of Inpatient Postpartum Care.	Ziabakhsh S, Fernandez R, Black B, Brito G.	2018	Canada	Peer-reviewed literature
Development of a tool to measure person-centered maternity care in developing settings: validation in a rural and urban Kenyan population.	Afulani PA, Diamond-Smith N, Golub G, Sudhinaraset M.	2017	Kenya	Peer-reviewed literature
Validation of the person-centered maternity care scale in India.	Afulani PA, Diamond-Smith N, Phillips B, Singhal S, Sudhinaraset M.	2018	India	Peer-reviewed literature
Person-centred maternity care in low-income and middle-income countries: analysis of data from Kenya, Ghana, and India.	Afulani PA, Phillips B, Aborigo RA, Moyer CA.	2019	Multi-country (Kenya, Ghana, India)	Peer-reviewed literature
Measuring Patient Experiences in Primary Health Care.	Wong ST, Haggerty J.	2013	Multi-country	Grey literature
Standards for improving quality of maternal and newborn care in health facilities.	World Health Organization	2016	Multi-country	Grey literature

Recommendations for Indicators for Newborns at Risk or with Complications	Every Newborn Action Plan	2022	Multi-country	Grey literature

Step 9. Sharing of provision of care proxy indicators and experience of care scale and additional items with the steering committee

We shared the provision of care proxy indicators and experience of care scale and additional items with the steering committee members via email. We also presented at a steering committee meeting and had a discussion around our selected indicators. There were requests that we further investigate the feasibility of our selected indicators.

Step 10. Feasibility of provision of care proxy indicators and experience of care scale and additional items

In response to the steering committee presentation discussions around the feasibility of implementing our selected indicators, we looked at the QED catalogue to compile information around potential data sources and methods for our selected indicators. We also checked the SPA Maternal and Newborn Health indicators for resubmission to see if any of our selected indicators were among their recommended indicators. Two of our provision of care indicators, essential newborn care and neonatal resuscitation, were recommended for retention as core indicators by the SPA resubmission. Indicators for vital sign assessment for women, fetal heart measurement and administration of PMTCT were recommended for removal from the core module, but for consideration for an optional module. As we have noted above, the 13-item PCMC scale has been recommended for retention for the SPA resubmission. The following experience of care items have also been recommended for retention: companion of choice, non-official payment, additional items. pre-discharge counselling, retained for non-payment of medical bill, family supportive environment and rooming-in. Finally, we checked the current version of the SPA and the HHFA to determine which components of our selected indicators are captured by these tools.

Table S14. Feasibility of provision of care indicators*

Indicator definition and number	Potential Data Sources or Methods listed in the QED catalogue	Information (indicator, definition, data collection method) for indicators recommended for retention by SPA resubmission	Data Collection Method for indicators SPA recommends removing from core module but consideration for optional module	Components of indicator captured in the SPA 2022	Components of indicator captured in HHFA
1. % women who gave birth in the health facility whose blood pressure, pulse, temperature, vaginal examination, and fetal heart sounds were taken during labour, childbirth, and the early postpartum period.‡	RIS for blood pressure, pulse, and temperature		Data collection Method: Record review. Recommend a review of data elements in charts/registers as part of the SPA planning process in each country. The Register/Chart Review module should only be undertaken in countries where the minimal data elements are available and of sufficient quality.	only asks about components during ANC and FP visit. Asks health care provider if they took client measurements. No mention of vaginal examination.	only has blood pressure Q for ANC.
2. % newborns who received all four elements of essential newborn care: immediate and thorough drying, immediate skin-to-skin contact, delayed cord clamping and initiation of breastfeeding in the first hour.‡	No potential data source listed; but other studies suggest some aspect of routine care is capturable through RIS.	Indicator: Essential newborn care: % simulated immediate care after birth for mother and newborn meeting minimal standard<br><br>Definition: Simulated immediate care after birth for mother and newborn meeting minimal standard:<br>- immediate and thorough drying and skin-to-skin;<br>- prophylactic uterotonic;<br>- delayed cord clamping;<br>- put to breast soon after birth<br>This indicator measures provider competency to provide integrated essential early newborn care and PPH prevention, Statements 1.1 and 1.3 in the WHO standards for improving quality of maternal and newborn care in health facilities(WHO 2016).		asks whether the facility routinely observes skin-to-skin, drying + wrapping newborns, initiation of breastfeeding within the first hour.	asks if the practices are expected to be implemented for all newborns. Asks whether providers have received training around essential newborn care in the last 2 years.

		Data Collection: Simulation. Tripathi and colleagues have validated a short index to measure quality of care at or immediately following delivery. The index has been used in health facilities Kenya, Madagascar, and Tanzania, including Zanzibar. This index and associated observation modules could be adapted for simulated cases. In addition, the SPA has an optional tool.			
3. % women with severe pre-eclampsia or eclampsia in the health facility who received magnesium sulfate.‡	RIS			asks if magnesium sulfate is available. Asks if parenteral administration of magnesium sulfate for management of pre-eclampsia and eclampsia has been carrie out in the past 12 months	
4. % women with severe hypertension in pregnancy in the health facility who received the recommended antihypertensives.	No corresponding QED indicator.			asks whether antihypertensives are available at the site	
5. % women with post-partum haemorrhage due to atony in the health facility who received therapeutic uterotonic drugs. ‡	RIS for "% women administered immediate postpartum uterotonic" does		Indicator: Uterotonics: % women administered prophylactic uterotonic soon after birth	asks if oxytocin or other injectable uterotonic is available at the site	

	not specify cause of PPH		Data Collection Method: Record review. Recommend a review of data elements in charts/registers as part of the SPA planning process in each country. The Register/Chart Review module should only be undertaken in countries where the minimal data elements are available and of sufficient quality.		
6. % women in the health facility with post-partum haemorrhage due to a retained placenta for whom manual removal of the placenta was performed.	No corresponding QED indicator			asks whether manual removal of placenta has been performed in the last 3 months	asks whether manual removal of placenta has been performed in the past 12 months
7. % women in the health facility with prolonged and/or obstructed labour who gave birth by caesarean section. ‡	RIS			asks whether cesarean delivery has been performed in the last 3 months	asks whether caesarean has been performed in the past 12 months (does not specify whether it was due to prolonged and/or obstructed labour)
8. % women with delayed second stage of labour who underwent instrumental vaginal	No corresponding QED indicator				

birth or c-section (disaggregate by mode of delivery).					
9. % women in the health facility with confirmed delay in progress of the first stage of labour who received oxytocin for augmentation.	No corresponding QED indicator			asks whether oxytocin is available at the site	asks whether administration of uterotonic medicine (ex. Oxytocin) was available at all times in the past 3 months
10. % newborns who receive any positive pressure ventilation using any device (most commonly bag and mask). ‡	RIS	Indicator: Neonatal resuscitation: % simulated neonatal resuscitation cases meeting a minimum standard<br><br>Definition: Indicator of health provider competency for the management of neonatal asphyxia, corresponding to Standard 1.5 (WHO 2016)<br><br>Data Collection: Simulation. COP WG recommends adapting existent neonatal resuscitation simulation tools (e.g., Helping Babies Breathe).		asks whether self-inflating bag and mask are available at the site	asks whether unit has a resuscitation bag and mask for preterm and term infants. In another section, asks if neonatal resuscitation with bag and mask has been carried out in the past 12 months
11. % women with preterm pre-labour rupture of membranes who gave birth in the health facility who received prophylactic antibiotics.	No potential data source listed				there are questions about administration of antibiotics, but does not specify reason for administration

12. % women who delivered between 24 and 34 weeks gestational age who received at least one dose of ACS. ‡	RIS				asks whether the facility has administered corticosteroids for preterm labour to the mother to prevent respiratory complications in the newborn in the last 12 months
13.% live-born low-birth-weight (<2500g) newborns born in the health facility who are initiated on kangaroo mother care (KMC)** or admitted to a KMC unit if a separate unit exists. ‡	RIS			asks whether kmc has been performed at least once in the past 3 months	asks whether kmc is ever provided for premature or underweight newborns. Asks whether kmc has been provided at any time in the past 3 months.
11.% women who gave birth in the health facility with preterm pre-labour rupture of membranes who received prophylactic antibiotics.§	No potential data source listed				asks whether they have administered antibiotics
14.% women who gave birth in the health facility with signs of infection treated with appropriate antibiotics. ‡	RIS				asks whether they have administered antibiotics

15.% newborns identified as cases of possible serious bacterial infection in outpatient settings or clinically suspected sepsis in inpatient settings who received at least two days of appropriate injectable antibiotics. ‡	RIS			asks whether injectable antibiotics are available at the site	asks whether they have administered antibiotics
16. % sick, preterm or small newborns who could not be managed at the health facility who were transferred to an appropriate level of care within 1 h of a decision, accompanied by a health care professional and a completed standardized referral note. ‡	RIS, admin				asks whether the facility uses a pre-printed referral form when patients are referred elsewhere
17. % pregnant or postnatal women who could not be managed at the health facility who were transferred to a higher-level facility for childbirth or further management without delay, accompanied by a health care	RIS, admin				asks whether the facility uses a pre-printed referral form when patients are referred elsewhere

professional and a completed standardized referral note. ‡					
18. % pregnant and postnatal women and newborns who were referred with appropriate emergency transport (disaggregate by pregnant woman, postnatal woman, newborn).	No potential data source listed.				asks whether facility has access to a functional ambulance or other vehicle, and whether the vehicle and driver are available 24 hours
19. % referred women and newborns seen at the referring facility who received timely care at the referral facility (disaggregate by women, newborns).	No potential data source listed.				
20. % women living with HIV who delivered in the health facility and received appropriate prophylaxis to prevent mother-to-child transmission and antiretroviral therapy.	No corresponding QED indicator.		Data Collection Method: Record review/chart sampling for data collection.		

Note: We considered core references as the WHO Standards, QED catalogue and ENAP metric recommendations.

*All indicators should be measured based on a specified time period.

†Kangaroo mother care is defined as care of preterm infants carried skin-to-skin with the mother. Key features include continuous skin-to-skin contact with the mother, exclusive breastfeeding, and early discharge from hospital in the kangaroo position with frequent home visits by health workers.
‡ Indicators identified as collectable through routine information systems.
§ Duplicate indicator that was categorized under two WHO Standards.

Table S15. Feasibility of experience of care indicators

**Original 30-item PCMC scale**	**Recommended for retention by SPA resubmission**
**Supportive care**	***13-item PCMC will be included in SPA
1. How did you feel about the amount of time you waited? Would you say it was very short, somewhat short, somewhat long, or very long?	
2. Were you allowed to have someone you wanted to stay with you during labor?	Indicator: Companion of choice: % women who wanted and had a companion of their choice supporting them in the health facility:<br>(1) during labor<br>(2) during childbirth<br>(3) after birth until time of discharge<br><br>Definition: Proportion of women who wanted and had a companion supporting them during [labour] [childbirth] in the health facility<br><br>Data Collection Method: Client Exit Interview<br><br>Note: Split into 3 indicators
3. Were you allowed to have someone you wanted to stay with you during the delivery?	
4. When you needed help, did you feel the doctors, nurses, or other staff at the facility paid attention?	
5. Did the doctors and nurses at the facility talk to you about how you were feeling?	
6. Did the doctors, nurses or other staff at the facility support your anxieties and fears?	
7. Do you feel the doctors or nurses did everything they could to help control your pain?	
8. Did you feel the doctors, nurses, or other staff at the facility took the best care of you?	

9. Did you feel you could completely trust the doctors, nurses, or other staff at the facility with regards to your care?	
10. Thinking about the wards, washrooms, and the general environment of the health facility, will you say the facility was very clean, clean, dirty, or very dirty?	
11. Do you think there was enough health staff in the facility to care for you?	
12. Thinking about the labor and postnatal wards, did you feel the health facility was crowded? (revised wording: Did you feel the place you gave birth was crowded during your birth stay? (e.g., not enough beds, moved from room to room, being in triage a long time)	
13. In general, did you feel safe in the health facility?	
14. Was there water in the facility?	
15. Was there electricity in the facility?	
**Dignity and respect**	
16. Did the doctors, nurses, or other staff at the facility treat you with respect?	
17. Did the doctors, nurses, and other staff at the facility treat you in a friendly manner?	
18. During examinations in the labor room, were you covered up with a cloth or blanket or screened with a curtain so that you did not feel exposed?	
19. Do you feel like your health information was or will be kept confidential at this facility?	
20. Did you feel the doctors, nurses, or other health providers shouted at you, scolded, insulted, threatened, or talked to you rudely?	
21. Did you feel like you were treated roughly… like pushed, beaten, slapped, pinched, physically restrained, or gagged?	
**Communication and autonomy**	
22. During your time in the health facility did the doctors, nurses, or other health care providers introduce themselves to you when they first came to see you?	
23. Did the doctors, nurses, or other health care providers call you by your preferred name?	
24. Did you feel like the doctors, nurses, or other staff at the facility involved you in decisions about your care?	
25. Did the doctors and nurses explain to you why they were doing examinations or procedures on you?	
26. Did the doctors and nurses explain to you why they were giving you any medicine?	
27. Did the doctors, nurses, or other staff at the facility ask your permission/consent before doing procedures and examinations on you?	
28. During the delivery, do you feel like you were able to be in the position of your choice?	

29. Did the doctors, nurses, or other staff at the facility speak to you in a language or in terms you could understand.	
30. Did you feel you could ask the doctors, nurses or other staff at the facility any questions you had?	
Notes: Items in the PCMC scale are measured on a 4-point scale (with most as 0: No, never; 1: Yes, a few times; 2: Yes most of the time; 3: Yes, all the time). The responses are added to create PCMC and sub-scale scores which can be standardized to range from 0 to 100. The items highlighted in yellow correspond to the shorter 13-item PCMC scale.	
**Additional EOC indicators/questions identified from review not captured by PCMC scale questions**	
1. The proportion of all women discharged from the labour and childbirth area of the facility who received written and verbal information and counselling on the following elements before discharge: nutrition and hygiene, birth spacing and family planning, exclusive breastfeeding and maintaining lactation, keeping their baby warm and clean, communication and play with the baby, danger signs for the mother and newborn and where to go in case of complications.	Indicator:<br>Proportion of women who received pre-discharge counselling for the mother and the baby in a given period.<br><br>Definition: Number of women who received pre-discharge counselling on each element on the list after childbirth and before discharge on: FP, exclusive breastmilk feeding, nutrition, IFA supplements, emotional well-being of mother, danger signs for the mother, newborn danger signs, birth registration, newborn immunization, stimulation and play with child, when to return to the health facility for mother and newborn.<br><br>Data collection Method: Client Exit Interview
2. The proportion of all women who gave birth in the health facility who reported that they were given the opportunity to discuss their concerns and preferences.	
3. The proportion of women who reported that they were told different things by different care providers about their health that led to confusion.	

4. The proportion of all women who gave birth in the health facility who reported that health care staff showed good knowledge of the women's history and the care that had been given to date.	
5. The proportion of women who felt that their newborn's health information was or would be kept confidential at the facility.	
6. Proportion of women who reported that health providers sexually harassed them or made sexual advances (for example, inappropriate touching or sexual comments that make them feel uncomfortable)	
7. Proportion of newborns who were maltreated.	
8. Proportion of mothers not cleaned after birth and third stage of labour	
9. Proportion of women who were instructed to clean up blood, urine, faeces or amniotic fluid.	
10. Proportion of newborns who had prompt removal of soiled wrapper or diaper and cleaning of urine and faeces	
11. Proportion of women not denied care (e.g., refused care for any reason)	
12. Proportion of women not detained at facilities due to lack of payment	Indicator: Women retained for non-payment of medical bill: % women retained in maternity for non-payment of medical bill<br><br>Definition: Proportion of women retained in maternity for non-payment of medical bill.<br><br>Data Collection Method: Client Exit Interview
13. The proportion of women who gave birth in the health facility who were aware of the existence and location of a complaints box.	
14. The proportion of all women in the health facility who made a complaint whose complaints were acted upon without repercussions.	
15. The proportion of procedures in the health facility that require written consent for which there is an associated record of consent signed by the woman or a family member.	

16. Proportion of carers in the health facility who report having received information about the care plan for their newborn.	
17. % of parents who felt that the staff at the local referring hospital explained the reason for transfer of their baby (only applies if baby was transferred from another unit).	
18. Proportion of small and sick newborns in the health facility whose carers reported participating in their newborn's care.	
19. Acceptance of various family types and parental role arrangements.	
20. % of mothers (carers) who report they were supported in family-centered care (facility allows companion/ family member, there is place for family member to sleep, place for family member to eat, place for family member to bathe).	Indicator: Family supportive environment: % women reporting a family-supportive environment (facility policy allows companion/ family member, there is place for family member to sleep)<br><br>Definition: Facility policy allows companion/family member, there is place for family member to sleep<br><br>Data Collection Method: Client Exit Interview<br><br>Note: Refine indicator wording
21. % of women who reported having direct access to the bathroom in the room they were lying after giving birth	
22. % of women who reported that the hospital, clinic, or healthcare provider(s) office was accessible given their needs (ex. specialized equipment, extra space).	
23. The proportion of all women undergoing bereavement or an adverse outcome who received additional emotional support from health facility staff	
24. Proportion of small and sick newborns who received appropriate developmental supportive care during their stay in the health facility.	
25. How did you feel about your length of stay at the hospital after giving birth?	
26. Proportion of women who reported being separated from their baby without medical indication.	Indicator: Women with access to her baby at all times (rooming-in): % of

	women who report they had access to her baby at all times<br><br>Definition: Women who had access to their babay at all times while in the facility<br><br>Data Source: Client Exit Interview
27. Proportion of women who were encouraged and/or able to mobilize.	
28. The proportion of all healthy mothers on postnatal wards or areas in the health facility who received breastfeeding counselling and support from a skilled health care provider.	Indicator: Breastfeeding support: % of women of in the health facility who have received support for breast milk feeding<br><br>Definition: Women who received support for breast milk feeding<br><br>Data Source: Client Exit Interview:<br><br>Note: Harmonize with nutrition group (BFHI)

Section 5. Gap areas

Patient safety and provider experience were identified as two gap areas of the WHO Standards framework by members of the Steering Committee when the quality of care scoping review project was first presented. To address these potential gaps, a brief review of literature related to patient safety and provider experience was conducted.

Resource identification

Resources for this review were identified in 4 ways:

1) Relevant guidance documents

- Relevant guidance documents on patient safety and provider experience were compiled from a targeted web search of UN agencies and leading international medical associations such as FIGO.

2) Systematic review of the literature on patient safety.

- The search strategy was modeled after the systematic review conducted for experience of care. Since the experience of care search strategy captured a comprehensive list of experience of care indicators, we narrowed down the search terms and added specific terms related to patient safety. The search was conducted using Pubmed and the search period was January 1, 2019 to April 1, 2022.
- Relevant articles were extracted from the sources identified in the search that had been published in the last decade

  (maternal health[tiab] OR maternal service*[tiab] OR maternity care[tiab] OR maternal care[tiab] OR maternity service*[tiab] OR Maternal Health[mesh] OR Maternal Health Services[mesh] OR newborn health[tiab] OR newbornservice*[tiab] OR newborn care[tiab] OR infant, newborn[mesh] OR NewbornHealth Services[tiab] OR infant health[tiab] or infant service*[tiab] OR infantcare[tiab] OR infant service*[tiab] OR infant health services[tiab] OR neonatehealth[tiab] OR neonatal health[tiab] OR neonatal service*[tiab] OR neonatalcare[tiab] OR neonate care[tiab] OR neonat* service*[tiab] OR neonat* healthservice*[tiab] AND (discrimination[tiab] OR disrespect[tiab] OR abuse[tiab] OR mistreatment[tiab] OR safety[tiab] OR harmful[tiab])

3) Systematic review of the literature on provider experience

- The search strategy for provider experience was modeled in the same way as the search strategy for patient safety. Search terms from the experience of care search were narrowed down and specific terms related to provider experience were added.The search was conducted using Pubmed and the search period was January 1, 2019 to April 1, 2022.
- Relevant articles were extracted from the sources identified in the search that had been published in the last decade

maternal health[tiab] OR maternal service*[tiab] OR maternity care[tiab] OR maternal care[tiab] OR maternity service*[tiab] OR "Maternal Health"[mesh] OR"Maternal Health Services"[mesh] OR newborn health[tiab] OR newbornservice*[tiab] OR newborn care[tiab] OR "infant, newborn"[mesh] OR"Newborn Health Services"[tiab] OR infant health[tiab] or infant service*[tiab]OR infant care[tiab] OR infant service*[tiab] OR infant health services[tiab] OR neonate health[tiab] OR neonatal health[tiab] OR neonatal service*[tiab] ORneonatal care[tiab] OR neonate care[tiab] OR neonat* service*[tiab] OR neonat*health service*[tiab] AND (Experience [tiab] OR "Provider Experience"[tiab])

4) Scoping review indicators

- Lastly, indicators related to patient safety and provider experience that had been identified during the scoping review and categorized under WHO quality statements, were extracted and included in the analysis.

Results

Patient Safety

There are no additional indicators to be added to address patient safety. The WHO quality statements 11.9 and 5.2 appropriately capture these measures for safety indicators (WHO, 2016). Most research surrounding patient safety relates to disrespect and abuse. Other measures include maltreatment, mistreatment, disrespect, quality of care, respectful care, unnecessary and harmful practices, and violence. Patient safety might also include human resources and infrastructure (i.e., minimum staff requirements, WASH, and electricity). These related measured will be explored further under the readiness review of the literature.

<u>Provider Experience</u>

Global consensus documents do not provide a specific scale for measuring provider experience. However, there are scales measuring provider stress and burnout (Shirom et al.,1989; Maslach et al., 1996). Existing provider experience indicators within our review fall under WHO quality statement 4.2 (WHO, 2016). Although, there remains a gap area in the lack of consensus surrounding a comprehensive provider experience scale. It is important that this gap is addressed to better capture the experience of providers within the EmONC context.

Next steps

- Revision of indicators/methods document
- Presentation to the quality of care workstream, end of July
- Harmonization with the referral study and with the ENAP newborn indicator recommendations
- Broader consultation with the maternal community and a human centered design process
- Revision and finalization of the methods document and preparation of paper for peer-review
- Registration of the scoping review and submission to peer-review journal
- Finalized set of indicators sent to Steering Committee and AMDD for integration into handbook development process

Table S16. Quality of Care Working Group member affiliations and locations

Name	Affiliation	Location
Alison Morgan	Global Financing Facility, World Bank	United States
Allisyn Moran	World Health Organization	Geneva
Andreea Creanga	The Johns Hopkins University	United States
Blerta Maliqi	World Health Organization	Geneva
Caitlin Warthin	Averting Maternal Deaths and Disabilities, Columbia University	United States
Catherine Breen Kamkong	United Nations Population Fund	Thailand
Dilys Walker	University of California San Francisco	United States
Patricia Bailey	Averting Maternal Deaths and Disabilities, Columbia University	United States
Jean-Pierre Monet	United Nations Population Fund	United States
Kathleen Hill	Jhpiego	United States
Lenka Benova	Institute of Tropical Medicine	Belgium
Louise-Tina Day	London School of Tropical Medicine	United Kingdom
Lynn Freedman	Averting Maternal Deaths and Disabilities, Columbia University	United States
Moise Muzigabe	World Health Organization	Geneva

Samantha Lobis	Averting Maternal Deaths and Disabilities, Columbia University	United States
Sodzi Sodzi-Tettey	Institute for Healthcare Improvement	Ghana
Tedbabe Degefie Hailegebriel	United Nations Children's Fund	United States

Table S17. Quality of Care Steering Committee member affiliations and locations

**Name**	**Affiliation**	**Location**
Lynn Freedman	Averting Maternal Deaths and Disabilities, Columbia University	United States
Patricia Bailey	Averting Maternal Deaths and Disabilities, Columbia University	United States
Samantha Lobis	Averting Maternal Deaths and Disabilities, Columbia University	United States
Isabelle Moreira	Averting Maternal Deaths and Disabilities, Columbia University	Senegal
Caitlin Warthin	Averting Maternal Deaths and Disabilities, Columbia University	United States
Sarah Moxon	London School of Hygiene and Tropical Medicine	United Kingdom
Oona Campbell	London School of Hygiene and Tropical Medicine	United Kingdom
Michel Brun	United Nations Population Fund	United States

Jean-Pierre Monet	United Nations Population Fund	United States
Jennifer Requejo	United Nations Children’s Fund	United States
Tedbabe Degefie Hailegebriel	United Nations Children’s Fund	United States
Gagan Gupta	United Nations Children’s Fund	United States
Allisyn Moran	Wolrd Health Organization	United States
Sudha Sharma	CIWEC Hospital	Nepal
Jalemba Aluvaala	University of Nairobi / KEMRI-Wellcome Trust	Kenya
Patience Afulani	University of California-San Francisco	United States

References

Afulani, P. A., Altman, M. R., Castillo, E., Bernal, N., Jones, L., Camara, T., Carrasco, Z., Williams, S., Sudhinaraset, M., & Kuppermann, M. (2022). Adaptation of the Person-Centered Maternity Care Scale in the United States: Prioritizing the Experiences of Black Women and Birthing People. *Women's Health Issues*. https://doi.org/10.1016/j.whi.2022.01.006

Afulani, P. A., Buback, L., McNally, B., Mbuyita, S., Mwanyika-Sando, M., & Peca, E. (2020). A Rapid Review of Available Evidence to Inform Indicators for Routine Monitoring and Evaluation of Respectful Maternity Care. *Global Health: Science and Practice*, *8*(1), 125–135. https://doi.org/10.9745/GHSP-D-19-00323

Afulani, P. A., Diamond-Smith, N., Golub, G., & Sudhinaraset, M. (2017). Development of a tool to measure person-centered maternity care in developing settings: Validation in a rural and urban Kenyan population. *Reproductive Health*, *14*, 118. https://doi.org/10.1186/s12978-017-0381-7

Afulani, P. A., Diamond-Smith, N., Phillips, B., Singhal, S., & Sudhinaraset, M. (2018). Validation of the person-centered maternity care scale in India. *Reproductive Health*, *15*(1), 147. https://doi.org/10.1186/s12978-018-0591-7

Afulani, P. A., Feeser, K., Sudhinaraset, M., Aborigo, R., Montagu, D., & Chakraborty, N. (2019). Toward the development of a short multi-country person-centered maternity care scale. *International Journal of Gynecology & Obstetrics*, *0*(0). https://doi.org/10.1002/ijgo.12827

Afulani, P. A., Phillips, B., Aborigo, R. A., & Moyer, C. A. (2019). Person-centred maternity care in low-income and middle-income countries: Analysis of data from Kenya, Ghana, and India. *The Lancet. Global Health*, *7*(1), e96–e109. https://doi.org/10.1016/S2214-109X(18)30403-0

Donabedian, A. (1988). The quality of care. How can it be assessed? *JAMA: The Journal of the American Medical Association*, *260*(12), 1743–1748.

Larson, E., Sharma, J., Nasiri, K., Bohren, M.A., & Tunçalp, Ö. (2020). Measuring experiences of facility-based care for pregnant women and newborns: a scoping review. *BMJ Global Health,* 5: e003368. doi:10.1136/bmjgh-2020-003368

Maslach, C., Jackson, S. E., & Leiter, M. P. (1996). *Maslach burnout inventory manual.* Palo Alto, Calif. 577 College Ave., Palo Alto 94306:Consulting Psychologists Press.

Özşahin, Z., Altiparmak, S., Aksoy Derya, Y., Kayhan Tetik, B., & Inceoğlu, F. (2021). Turkish validity and reliability study for the person-centered maternity care scale. *The Journal of Obstetrics and Gynaecology Research*, *47*(9), 3211–3222. https://doi.org/10.1111/jog.14913

Marsh, A.D., Muzigaba, M., Diaz, T., Requejo, J., Jackson, D., Chou, D., et al. (2020). Effective coverage measurement in maternal, newborn, child, and adolescent health and nutrition, progress, future prospects, and implications for quality health systems. *The Lancet Global Health*, *8*(5), e730-e736. https://doi.org/10.1016/S2214-109X(20)30104-2

Moxon, S. G., Guenther, T., Gabrysch, S., Enweronu-Laryea, C., Ram, P. K., Niermeyer, S., Kerber, K., Tann, C. J., Russell, N., Kak, L., Bailey, P., Wilson, S., Wang, W., Winter, R., Carvajal-Aguirre, L., Blencowe, H., Campbell, O., & Lawn, J. (2018). Service readiness for inpatient care of small and sick newborns: What do we need and what can we measure now? *Journal of Global Health*, *8*(1), 010702. https://doi.org/10.7189/jogh.08.010702

Perin, J., Mulick, A., Yeung, D., Villavicencio, F., Lopez, G., & Strong, K.L. (2021). Global, regional, and national causes of under-5 mortality in 2000-19: an unpdated sysematic analysis with implications for the Sustainable Development Goals. Child & Adolescent Health, 6(2), 106-115. DOI: https://doi.org/10.1016/S2352-4642(21)00311-4

QED Network. (2019). Quality of care for maternal and newborn health: A monitoring framework for network countries. https://www.who.int/publications/m/item/quality-of-care-for-maternal-and-newborn—a-monitoring-framework-for-network-countries.

Sacks, E. (2017). Defining disrespect and abuse of newborns: a review of the evidence and an expanded typology of respectful maternity care. *Reproductive Health,* 14:66. DOI 10.1186/s12978-017 -0326-1

Say, L., Chou, D., Gemmill, A., Tuncalp, O., Moller, A., & Daniels, J. (2014). Global causes of maternal death: a WHO systematic analysis. Global Health, 2(6), e323-e333. DOI: https://doi.org/10.1016/S2214-109X(14)70227-X.

Shirom, A. (1989). Burnout in work organizations. In C. L. Cooper & I. T. Robertson (Eds.), *International review of industrial and organizational psychology 1989* (pp. 25–48). John Wiley & Sons

SPA Maternal Newborn Health Working Group. (2021, June 1). *Maternal and Newborn Health Indicators for Resubmission*.

Rishard, M., Fahmy, F. F., Senanayake, H., Ranaweera, A. K. P., Armocida, B., Mariani, I., & Lazzerini, M. (2021). Correlation among experience of person-centered maternity care, provision of care and women's satisfaction: Cross sectional study in Colombo, Sri Lanka. *PLOS ONE*, *16*(4), e0249265. https://doi.org/10.1371/journal.pone.0249265

Tunçalp, Ö., Were, W., MacLennan, C., Oladapo, O., Gülmezoglu, A., Bahl, R., Daelmans, B., Mathai, M., Say, L., Kristensen, F., Temmerman, M., & Bustreo, F. (2015). Quality of care for pregnant women and newborns—The WHO vision. *BJOG: An International Journal of Obstetrics & Gynaecology*, *122*(8), 1045–1049. https://doi.org/10.1111/1471-0528.13451

Wong ST, Haggerty JL. Measuring Patient Experiences in Primary Health Care. Heal Serv Policy Res. 2013;(May):1–34

WHO. (2016). *Standards for improving quality of maternal and newborn care in health facilities*. http://www.who.int/maternal_child_adolescent/documents/improving-maternal-newborn-care-quality/en/
